# Diagnostic accuracy of depression questionnaires in adult patients with diabetes: A systematic review and meta-analysis

**DOI:** 10.1371/journal.pone.0218512

**Published:** 2019-06-20

**Authors:** Johanna W. de Joode, Susan E.M. van Dijk, Florine S. Walburg, Judith E. Bosmans, Harm W.J. van Marwijk, Michiel R. de Boer, Maurits W. van Tulder, Marcel C. Adriaanse

**Affiliations:** 1 Department of Health Sciences, Amsterdam Public Health Research Institute, Vrije Universiteit Amsterdam, Amsterdam, The Netherlands; 2 Knowledge Institute of Medical Specialists, Utrecht, The Netherlands; 3 Department of Primary Care and Public Health, University of Brighton, Brighton, United Kingdom; 4 Brighton and Sussex Medical School, Watson Building House, University of Brighton, Brighton, United Kingdom; University of Mississippi Medical Center, UNITED STATES

## Abstract

**Background:**

Comorbid depression is common among patients with diabetes and has severe health consequences, but often remains unrecognized. Several questionnaires are used to screen for depression. A systematic review and meta-analysis regarding the diagnostic accuracy of depression questionnaires in adults with diabetes is unavailable. Our aim was to conduct a systematic review and meta-analysis to evaluate the diagnostic accuracy of depression questionnaires in adults with type 1 or type 2 diabetes.

**Methods:**

PubMed, Embase and PsycINFO were searched from inception to 28 February 2018. Studies were included when the diagnostic accuracy of depression questionnaires was assessed in a diabetes population and the reference standard was a clinical interview. Data extraction was performed by one reviewer and checked by another. Two reviewers independently conducted the quality assessment (QUADAS-2). Diagnostic accuracy was pooled in bivariate random effects models. The main outcome was diagnostic accuracy, expressed as sensitivity and specificity, of depression questionnaires in an adult diabetes population. This study is reported according to PRISMA-DTA and is registered with PROSPERO (CRD42018092950).

**Results:**

A total 6,097 peer-reviewed articles were screened. Twenty-one studies (N = 5,703 patients) met the inclusion criteria for the systematic review. Twelve different depression questionnaires were identified, of which the CES-D (n = 6 studies) and PHQ-9 (n = 7 studies) were the most frequently evaluated. Risk of bias was unclear for multiple domains in the majority of studies. In the meta-analyses, five (N = 1,228) studies of the CES-D (≥16), five (N = 1,642) of the PHQ-9 (≥10) and four (N = 822) of the algorithm of the PHQ-9 were included in the pooled analysis. The CES-D (≥16) had a pooled sensitivity of 85.0% (95%CI, 71.3–92.8%) and a specificity of 71.6% (95%CI, 62.5–79.2%); the PHQ-9 (≥10) had a sensitivity of 81.5% (95%CI, 57.1–93.5%) and a specificity of 79.7% (95%CI, 62.1–90.4%). The algorithm for the PHQ-9 had a sensitivity of 60.9% (95%CI, 52.3–90.8%) and a specificity of 64.0% (95%CI, 53.0–93.9%).

**Conclusions:**

This review indicates that the CES-D had the highest sensitivity, whereas the PHQ-9 had the highest specificity, although confidence intervals were wide and overlapping. The algorithm for the PHQ-9 had the lowest sensitivity and specificity. Given the variance in results and suboptimal reporting of studies, further high quality studies are needed to confirm the diagnostic accuracy of these depression questionnaires in patients with diabetes.

## Introduction

Depression among patients with diabetes is common and has severe health consequences. Depression is defined as severely depressed mood that persists for at least two weeks in combination with 5 of the symptoms (i.e. loss of pleasure, changes in sleep pattern, early rising, changes in appetite with weight loss/gain, feelings of guilt/worthlessness, low energy level, difficulty concentrating, nervousness, morning sadness)[1]. Comorbid depression is present in 12% to 19% of patients with type 1 and type 2 diabetes respectively[2]. The number of people suffering from both depression and diabetes is expected to rise sharply in the next decade[3, 4]. Comorbid depression is associated with a reduction in quality of life[1, 5], poorer self-care behavior[1, 6, 7], deterioration of glycemic control[1, 7, 8], and increased expenditure on health care costs[9, 10]. Moreover, patients with both diabetes and depression have more comorbidities[1, 7, 11] and show higher mortality rates[1, 7, 12] compared to diabetes patients without depression.

Although effective treatment options for depression in patients with diabetes are available[13, 14], comorbid depression may still be a problematic issue. Depression may remain unacknowledged and undiagnosed in more than half of the cases in both specialized diabetes centers[15] and non-specialized centers[16], thereby possibly missing appropriate intervention and treatment. The main reasons that patients and health care professionals may not discuss depression as an issue include the focus on somatic symptoms and complications, undue normalization of depressive symptoms, and a lack of opportunity to discuss mental health in routine diabetes consultations[17]. Screening for depression is recommended in clinical guidelines[18–21] and various depression questionnaires are used for screening and diagnosing purposes[22–26]. These questionnaires are often based on the criteria of the Diagnostic and Statistical Manual of Mental Disorders III or IV (DSM-III or DSM-IV).

Some symptoms of depression (e.g., change in appetite, changes in weight, loss of energy and difficulties in concentrating) are also common in diabetes. This may result in an overestimation of depressive symptoms in diabetes patients and, higher scores on depression questionnaires, resulting in a higher false positive rate. To ensure existing depression screening questionnaires can be validly used in a population of diabetes patients, many of these have undergone psychometric testing in this specific population. Recently, a systematic review focusing on measurement properties (i.e. reliability, validity and responsiveness) of these questionnaires in a diabetes population was performed and found that, based on the current knowledge, the Centre for Epidemiological Studies Depression Scale (CES-D) is the best questionnaire for monitoring depressive symptoms[27]. However, screening purposes are related to other measurement properties (i.e. sensitivity and specificity) than monitoring purposes. The screening and diagnostic quality of a tool is determined by the diagnostic accuracy of a test, which is defined as “a test’s ability to discriminate between people with the target condition and those without” compared to a reference standard[28], such as a clinical interview for depression.

Roy et al. (2012) performed a systematic review of the literature in which they identified frequently used depression questionnaires in a diabetes population, and the corresponding sensitivity and specificity of these questionnaires. However, a meta-analysis and quality assessment were not included[29]. Practical recommendations regarding the use of specific tools could therefore not be made. Furthermore, the correlation between specificity and sensitivity was not taken into account[29], as recommended by the Cochrane Collaboration[28]. The aim of this study was to conduct a systematic review and meta-analysis to evaluate the diagnostic accuracy of depression questionnaires in adults with type 1 or type 2 diabetes.

## Materials and methods

### Design

This study is registered with PROSPERO, number CRD42018092950[30], and is reported according to the Preferred Reporting Items for a Systematic Review and Meta-analysis of Diagnostic Test Accuracy Studies (PRISMA-DTA) (S1 and S2 Tables)[31].

### Search strategy and study selection

PubMed, EMBASE and PsycINFO were searched from inception up to February 28, 2018. The search strategy consisted of terms for diabetes and depression (S3 Table). Terms about diagnostic accuracy and questionnaires were not included because clear terms for identifying diagnostic accuracy studies in databases are lacking[28, 32] and no studies should be missed. Studies were included when the diagnostic accuracy of depression questionnaires was measured in a diabetes population (i.e. at least 80% of the population had diabetes type 1 or 2) and the reference standard was a clinical interview. There were no language restrictions. Depression questionnaires are defined as questionnaires which are developed to measure depressive symptoms. Despite the fact that the World Health Organization-Five Well-Being (WHO-5) was originally developed for the assessment of subjective psychological well-being, it was included, because this questionnaire is widely used for measuring depression symptoms[33]. Duplicate records were removed according to the recommendations of Bramer et al.[34]. The titles and abstracts of peer-reviewed full articles were screened; comments, letters, editorials, book sections and theses were excluded.

Pairs of review authors independently assessed titles and abstracts to identify relevant articles. Full-texts were retrieved when both review authors agreed that studies were relevant or when consensus was not reached. Three review authors read the full-texts to judge study eligibility, independently. Disagreements were resolved by discussion, when consensus was not reached, a fourth reviewer made the final decision. Reference lists of included studies were screened for additional relevant studies by two review authors independently.

### Data extraction

Using a structured data extraction form, the following characteristics and data were extracted from included studies: sample size, age, gender, diabetes type, prevalence of depression in the sample, the country and setting in which the study was performed, depression questionnaire used, language, used thresholds with corresponding diagnostic accuracy properties (i.e. sensitivity, specificity, positive predictive value (PPV), negative predictive value (NPV), area under the curve (AUC)) and data to generate two-by-two-tables. Sensitivity of a questionnaire entails “the probability of a positive test given the presence of the disease”, while specificity entails “the probability of a negative test in those without the disease”[35]. Sensitivity and specificity of a questionnaire can be calculated at several thresholds. A threshold is defined as the sum score on a questionnaire that is the turning point between having a depression or not. The result of a screening questionnaire is used by clinicians to make decisions about further testing and therapy[18–20] and is used by researchers to make decisions about eligibility for participation in studies. For this reason, the depression questionnaire with the best diagnostic accuracy should be identified in particular for clinical practices and for research among patients with diabetes. The PPV is “the probability of the presence of disease in those with a positive test result” and the NPV is “the probability of absence of disease in those with a negative test result” [35]. The AUC in diagnostic accuracy studies is the area under the receiver operating characteristic (ROC) curve that reflects the inverse relationship between sensitivity and specificity at several thresholds. Data were extracted by one review author and checked by a second review author. The percentage of agreement for the data extraction was 0.94. Primary outcome of interest was diagnostic accuracy expressed as sensitivity and specificity of depression questionnaires in an adult diabetes population.

### Quality assessment

The quality assessment of included studies consisted of the following four domains according to the revised version of the Quality Assessment of Diagnostic Accuracy Studies (QUADAS-2): *Patient Selection*, *Index Test*, *Reference Standard* and *Flow and Timing*[36]. In this review, *Index Test* refers to the specific depression questionnaire evaluated. No signaling questions were added to or omitted from the QUADAS-2 format[36]. Interpretations of the signaling questions are described in S1 Text and S4 Table. All included studies were assessed for risk of bias in each domain and for applicability concerns in the first three domains. Risk of bias was judged as “low”, “high”, or “unclear”. Applicability concern is “the concern that the study does not fit in the review question” and was also judged “low”, “high” or “unclear”[36]. The quality assessment was independently performed by two review authors. The German and Spanish article were discussed with a native German and Spanish academic colleague, respectively. When consensus was not reached, a third review author decided.

### Data synthesis and statistical analysis

For the pooling of extracted data about sensitivity and specificity, at least three studies for each questionnaire with a corresponding threshold were needed. A bivariate random effects model was performed to adjust for the within- and between-study variance in sensitivity and specificity[37]. The method for the meta-analysis was based on the Stata manual of the Cochrane Handbook for Systematic Reviews of Diagnostic Test Accuracy[38]. Sensitivity and specificity were converted to two-by-two-tables to get data of true positives (TP), false positives (FP), true negatives (TN) and false negatives (FN). Then, data of the individual studies was plotted in a forest plot and a summary receiver operating characteristic (SROC) plot to illustrate the location and scatter of the data using RevMan (version 5.1). Analyses were conducted using the *metandi* option in StataSE (version 14). When the correlation between sensitivity and specificity could not be estimated, the *xtmelogit* option was used. These analyses resulted in a summary operating point (i.e. summary estimate for sensitivity and specificity) per questionnaire with 95% confidence region and 95% prediction region[38]. The 95% prediction region “illustrates the extent of statistical heterogeneity by depicting a region within which (assuming the model is correct) we have 95% confidence that the true sensitivity and specificity of a future study should lie”[39]. We aimed to investigate the source of heterogeneity between results using meta-regression and subgroup analysis. Prior to the analyses, variables that could lead to heterogeneity were selected. These were blinding of the reference standard, distribution of diabetes type, percentage of depression cases in the sample and setting. However, due to the low number of studies in the meta-analysis, it was not possible to perform meta-regression or subgroup analysis.

## Results

### Study inclusion and characteristics of included studies

**Fig 1:**
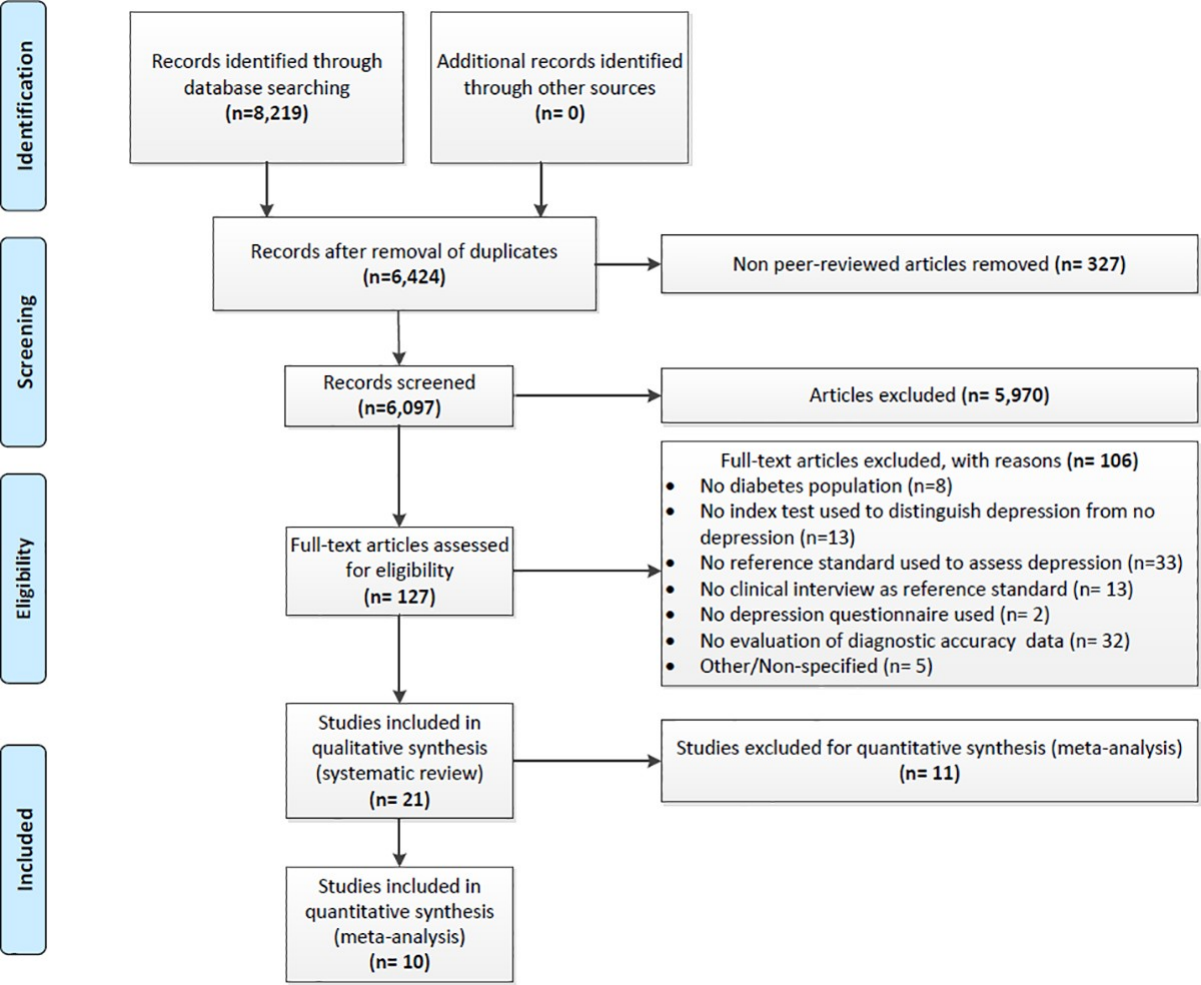
Flowchart of study selection process.

Fig 1 shows the study selection process in detail according to the PRISMA-DTA[31]. In the identification phase, 8,219 records were identified through database searching (S3 Table). No additional records were identified by screening of reference lists. In the screening phase, titles and abstracts of 6,097 full articles were screened. In the eligibility phase, 127 articles were selected for full-text retrieval, of which 106 were excluded. Reasons for exclusion are described in Fig 1. This resulted in the inclusion of 21 studies[40–60] for the systematic review (N = 5,703 patients). Of these, ten studies (N = 3,026 patients) were eligible for meta-analysis[43, 48, 49, 51, 53, 54, 56, 57, 59, 60] because at least three studies per threshold per questionnaire were needed.

Table 1 displays the characteristics of the included studies. Twelve different questionnaires were identified in the included studies, of which the CES-D and the Patient Health Questionnaire 9-item version (PHQ-9) were the most frequently evaluated. S5 Table presents the characteristics of the twelve questionnaires. In 19 studies consisted the study samples of patients with diabetes[40–46, 48–50, 52–60] and in two studies the diagnostic accuracy data was reported separately for patients with diabetes[47, 51]. Distribution of diabetes type differed between studies; from 100% diabetes type 1[44] to 100% diabetes type 2[40, 42, 43, 46, 48, 49, 51, 53, 55, 56, 59, 60]. Studies varied largely in sample sizes (range 65[41, 58]– 793[48]) and were conducted in different settings. Study samples differed in proportion of men (range 31.4[42]– 67.3%[48]), mean age (range 43.3[44]– 71.4[51] years) and prevalence of depression based on the clinical interview (range 3.5[44]– 43.2%[49]). S6 Table presents the extracted data regarding the diagnostic accuracy.

**Table 1:** Characteristics of included studies.

**Study**	**Questionnaire**	**Reference standard**	**Sample size**	Age, mean (SD)	**Male (%)**	DM1/DM2 (%)	Depression (%)^a^	**Setting**	**Country of study performance**	**Language of questionnaire**
**Ali (2013)[40]**	BDI	MINI	122	47.2 (9.4)	49.2	0/100	27.1	Hospital outpatient clinic	India	n.r.
**Awata (2007)[41]**	WHO-5	SCID-I	65	53.3 (11.0)	50.8	16.9/83.1	10.8	University hospital	Japan	Japanese
**Díaz-Rodríguez (2006) [42]**	CSDD	CIDI	528	63.0 (27–85) (median (range))	31.4	0/100	28.2	Primary care	Mexico	Spanish
**Fisher (2007)[43]**	CES-D	CIDI (Dx1)	506	57.8 (9.9)	43.0	0/100	4.0	Medical groups and diabetes education centers	USA	English, Spanish
**Fisher (2016)[44]**	PHQ-8	SCID	368	43.3 (17.7)	44.3	100/0	3.5	Hospital outpatient clinic	USA/Canada	English
**Hermanns (2006)[45]**	BDI; CES-D	CIDI or SCA	376	52.2 (14.3)	60.6	37.2/62.8	14.1	Hospital outpatient clinic	Germany	German
**Hsu (2014)[46]**	CUDOS	Clinical interview (n.s.)	212	62.6 (13.2)	45.8	0/100	17.0	University hospital outpatient clinic	Taiwan	Chinese
**Hyphantis (2015)[47]**	PHQ-9	MINI	194	70.1 (13.1)^**b**^	50.4^**b**^	n.r.	23.2	Accident and Emergency department	Greece	Greek
**Janssen (2016)[48]**	PHQ-9	MINI	793	62.4 (7.7)	67.3	0/100	7.7	Community based sample	The Netherlands	Dutch
**Khamseh (2011)[49]**	CES-D; PHQ-9	SCID	185	56.2 (9.6)	48.1	0/100	43.2	Specialized outpatient clinic	Iran	Persian
**Krille (2008)[50]**	WHO-5	Clinical interview (n.s.)	253	54.3 (14.0)	55.3	32.0/68.0	9.1	Hospital outpatient clinic	Germany	German
**Lamers (2008)[51]**	PHQ-9	MINI	365	71.4 (6.9)^**b**^	51.8^**b**^	0/100	n.r.	Primary care	The Netherlands	Dutch
**Lustman (1997)[52]**	BDI	DIS-Revised	172	48.1 (13.6)	52.3	n.r.	36.6	Community based sample	USA	English
**McHale (2008)[53]**	CES-D; DMI-10; HADS-D; SCAD	CIDI-SF	149; 147; 148; 149;	60.2 (12.0)	59.0	0/100	29.0	Hospital outpatient clinic	Australia	English
**Stahl (2008)[54]**	CES-D	SCAN	291	54.5 (13.3)^**b**^	n.r.	3.5/96.5^**b**^	17.5	Hospital diabetes centre	Singapore	English, Chinese, Malay
**Sultan (2010)[55]**	HADS-D; BDI-SF	MINI	298	59.4 (10.7)	55.0	0/100	10.1	Hospital outpatient clinic	France	French
**Twist (2013)[56]**	PHQ-9	SCAN(2.1)	368	55.7 (11.4)^**b**^	55.2^**b**^	0/100	22.8^**c**^	Primary care	United Kingdom	English
**Study**	**Questionnaire**	**Reference standard**	**Sample size**	**Age, mean** **(SD)**	**Male (%)**	**DM1/DM2** **(%)**	**Depression (%)**^**a**^	**Setting**	**Country of study performance**	**Language of questionnaire**
**Van Steenbergen-Weijenburg (2010)[57]**	PHQ-9	MINI	197	61.8 (13.7)	51.3	n.r.	18.8	Specialized outpatient clinic	The Netherlands	Dutch
**Yoshida (2009)[58]**	SDS	Clinical interview (n.s.)	65	53.6 (10.4)^**b**^	55.0^**b**^	18.6/81.4^**b**^	10.8	Hospital outpatient clinic	Japan	Japanese
**Zhang (2013)[59]**	PHQ-9	MINI	99	55.1 (9.5)^**b**^	59.2^**b**^	0/100	23.2	Hospital and community based diabetes centre	China	Chinese
**Zhang (2015)[60]**	CES-D	MINI	97	54.6 (9.5)^**b**^	58.7^**b**^	0/100	23.7	Hospital and community based diabetes centre	China	Chinese

BDI = Beck Depression Inventory; BDI-SF = Beck Depression Inventory-Short Form; CES-D = Centre for Epidemiological Studies Depression Scale; CIDI = Composite International Diagnostic Interview; CIDI (Dx1) = Composite International Diagnostic Interview Depression within the last month; CIDI-SF = Composite International Diagnostic Interview- Short Form; CSDD = Clinimetric Scale for the Diagnosis of Depression; DIS-Revised = National Institute of Mental Health Diagnostic Interview Schedule–Version IIIR; DM1 = Diabetes Mellitus type 1; DM2 = Diabetes Mellitus type 2; DMI-10 = Depression in the Medically Ill; DSM-IV = Diagnostic and Statistical Manual of Mental Disorders–Version IV; HADS-D = Hospital Anxiety and Depression Scale-Depression; MINI = Mini International Neuropsychiatric Interview; n.r. = not reported; n.s. = not specified; PHQ-8 = Patient Health Questionnaire 8-item version; PHQ-9 = Patient Health Questionnaire 9-item version; SCA = Standardized Clinical Assessment; SCAD = Silverstone Concise Assessment for Depression; SCAN(2.1) = Schedule for Clinical Assessment in Neuropsychiatry; SCID = Structured Clinical Interview for DSM Disorders; SCID-I = Structured Clinical Interview for DSM Disorders-Axis I; SD = standard deviation; SDS = Zung Self rating Depression Scale; USA = United States of America; WHO-5 = World Health Organization-Five Well-Being Index.

^a^ the percentage of depression cases in the sample based on the clinical interview

^b^ characteristics of the total cohort in the study; a subsample received the reference standard

^c^ not mentioned in the study, but based on created two-by-two-tables

### Quality assessment

Table 2 presents the results of the quality assessment regarding risks of bias and applicability concerns; explanations of decisions are listed in S4 Table. The risk of bias in the domain of *Patient selection* was low in the majority of studies[41, 42, 44, 45, 47, 48, 52, 54–56, 58–60]. The clinical interview was interpreted with knowledge of the scores on the depression questionnaire in two studies resulting in a high risk of bias in the domain of *Reference Standard*[51, 57]. In the majority of studies the procedure of testing patients was not clearly described resulting in an unclear risk of bias for the *Index test*[40, 43, 44, 46, 48, 50, 52, 55, 59, 60] and the *Reference Standard*[40, 42–44, 46, 48, 50, 52–54, 56, 59, 60]. In the domain *Flow and Timing* the risk of bias was either unclear[40, 42–44, 46–53, 59, 60] or high[41, 45, 54–58], because the procedure was not clearly described or the drop-out rates were high. Since appropriate index tests and reference standards were specified in inclusion criteria, all studies had low applicability concerns in domains *Index Test* and *Reference Standard*.

**Table 2:** Results of the quality assessment (QUADAS-2) of included studies.

First Author and Year	Risk of bias				Applicability Concerns		
	*Patient selection*	*Index test*	*Reference standard*	*Flow and Timing*	*Patient selection*	*Index test*	*Reference standard*
Ali (2013)[40]	☹	?	?	?	☹	☺	☺
Awata (2007)[41]	☺	☺	☺	☹	☺	☺	☺
Diaz-Rodriguez (2006)[42]	☺	☺	?	?	☺	☺	☺
Fisher (2007)[43]	☹	?	?	?	☹	☺	☺
Fisher (2016)[44]	☺	?	?	?	☺	☺	☺
Hermanns (2006)[45]	☺	☺	☺	☹	☺	☺	☺
Hsu (2014)[46]	☹	?	?	?	☹	☺	☺
Hyphantis (2015)[47]	☺	☺	☺	?	?	☺	☺
Janssen (2016)[48]	☺	?	?	?	☺	☺	☺
Khamseh (2011)[49]	☹	☺	☺	?	☹	☺	☺
Krille (2008)[50]	?	?	?	?	☺	☺	☺
Lamers (2008)[51]	☹	☺	☹	?	Λ	☺	☺
Lustman (1997)[52]	☺	?	☺	?	☹	☺	☺
McHale (2008)[53]	?	☺	?	?	☺	☺	☺
Stahl (2008)[54]	☺	☺	?	☹	☺	☺	☺
Sultan (2010)[55]	☺	?	☺	☹	☺	☺	☺
Twist (2013)[56]	☺	☺	?	☹	☹	☺	☺
v. Steenbergen-Weijenburg (2010)[57]	?	☺	☹	☹	☺	☺	☺
Yoshida (2009)[58]	☺	☺	?	☹	☺	☺	☺
Zhang (2013)[59]	☺	?	?	?	☺	☺	☺
Zhang (2015)[60]	☺	?	?	?	☺	☺	☺

☺ = Low Risk; ☹ = High Risk; ? = Unclear Risk

### Results of meta-analysis

**Fig 2:**
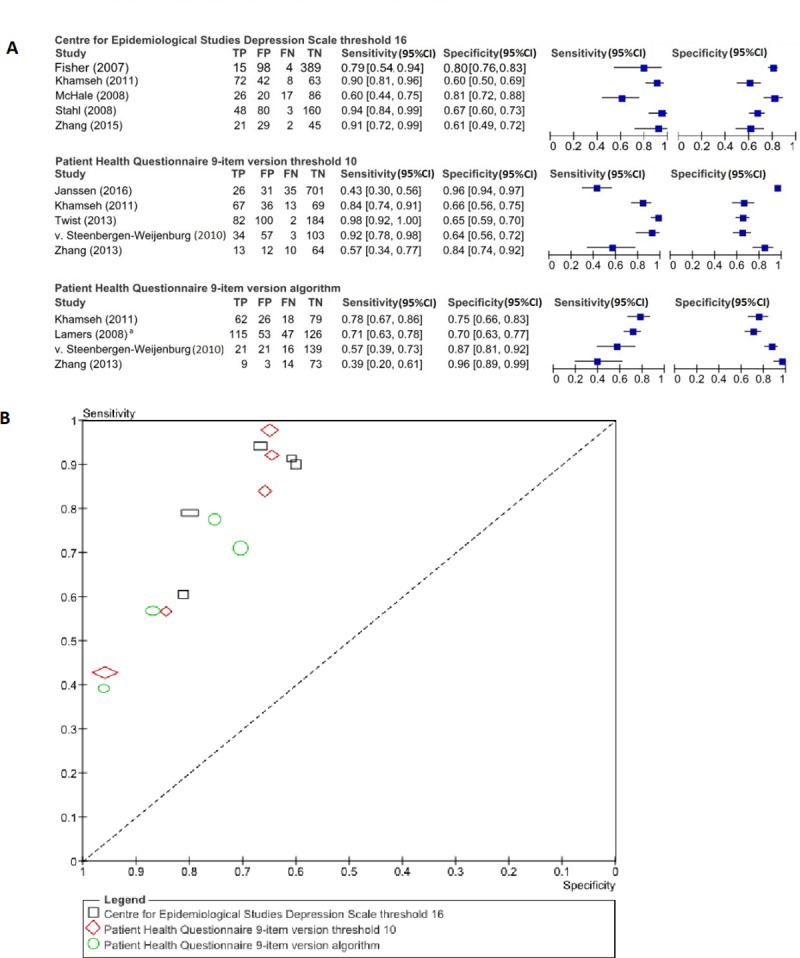
**Forest plots (A) and SROC plot (B) of the CES-D (≥16), PHQ-9 (≥10) and PHQ-9 (algorithm)** (A) 95%CI = 95% confidence interval; FN = false negatives; FP = false positives; TN = true negatives; TP = true positives. ^a^ two-by-two-table was obtained after correspondence with the author. (B) Each symbol represents a pair of sensitivity and specificity from a study and the size of symbols reflects the sample size of the study.

Only for the CES-D and the PHQ-9 there were at least three studies available for meta-analytical procedures. Data of the CES-D were pooled at a threshold of 16. For the PHQ-9 the data were pooled at a threshold of 10 and at the threshold according to the algorithm. The algorithm for the PHQ-9 is a specific threshold for identifying depression, which is defined in accordance with DSM-IV: five or more of the nine depressive symptoms criteria are present for at least more than half the days in the past two weeks and one of the symptoms is depressed mood or anhedonia[49]. The Forest plots (Fig 2A) and SROC plots (Fig 2B) contain the data that were pooled in the meta-analysis. Table 3 displays the summary operating points per questionnaire and S1 Fig displays these results visually in SROC plots. The CES-D (≥16) had a pooled sensitivity of 85.0% (95%CI, 71.3–92.8%) and a specificity of 71.6% (95%CI, 62.5–79.2%); the PHQ-9 (≥10) a sensitivity of 81.5% (95%CI, 57.1–93.5%) and a specificity of 79.7% (95%CI, 62.1–90.4%). Finally, the algorithm for the PHQ-9 had a sensitivity of 60.9% (95%CI, 52.3–90.8%) and a specificity of 64.0% (95%CI, 53.0–93.9%).

**Table 3:** Summary operating points of sensitivity and specificity by questionnaire.

Questionnaire	Threshold	N		Sensitivity (%) (95%CI)	Specificity (%) (95%CI)
		studies	participants		
CES-D	16	5	1,228	85.0 (71.3–92.8)	71.6 (62.5–79.2)
PHQ-9	10	5	1,642	81.5 (57.1–93.5)	79.7 (62.1–90.4)
	Algorithm	4	822	60.9 (52.3–90.8)	64.0 (53.0–93.9)

95%CI = 95% confidence interval; CES-D = Centre for Epidemiological Studies Depression Scale; PHQ-9 = Patient Health Questionnaire 9-item version

## Discussion

The results of the meta-analysis indicate that the CES-D (≥16) had the highest sensitivity and the PHQ-9 (≥10) had the highest specificity, although confidence intervals were wide and overlapping. The algorithm for the PHQ-9 had the lowest sensitivity and specificity.

In 2012, Roy et al. summarized the diagnostic accuracy of depression questionnaires among patients with diabetes in a systematic review in which 23 studies were included[29]. Only 7 of these studies were included in the current review because some studies did not meet our more strict inclusion criteria; especially the criterion that the reference standard should be a clinical interview was often not met. In the review of Roy et al. the correlation between sensitivity and specificity was not taken into account and there was no information on the exact thresholds[29]. Therefore, outcomes of the mean sensitivity and specificity from the review of Roy et al.[29] cannot be compared with the pooled outcomes of the current review.

Several reviews evaluated the diagnostic accuracy of depression questionnaires in other populations. A review from 2016 which evaluated the CES-D in the general population[61] reported a higher accuracy for the CES-D at a threshold of 20[61]. Unfortunately, this threshold was not used in any of the studies in this review. Similar to the current review, a meta-analysis from 2015 in the general population concluded that the diagnostic accuracy of the PHQ-9 at a threshold of 10 was better than for the algorithm[62]. However, the pooled specificity (94%) for the algorithm[62] was much higher than in the current review (64.0%). A possible explanation is that symptoms of depression and diabetes overlap, resulting in higher false positive and lower false negative rates at a certain threshold in patients with diabetes compared to people without diabetes. Two reviews in any population found comparable results on sensitivity (77%[62] and 78%[63]) and specificity (85%[62] and 87%[63]) as the current review (sensitivity of 81.5%; specificity of 79.7%).

### Strengths and limitations

To the best of our knowledge, this is the first systematic review that included a meta-analysis to evaluate the diagnostic accuracy of depression questionnaires among patients with diabetes type 1 or 2. Furthermore, a standardized tool (i.e. QUADAS-2) was used for the quality assessment and the meta-analysis was based on the Stata manual of Cochrane Handbook for Systematic Reviews of Diagnostic Test Accuracy. In addition, this systematic review and meta-analysis followed the recent PRISMA-DTA guidelines for transparent reporting.

However, there are some limitations. The number of studies per questionnaire in the meta-analysis was low (maximum of 5) because the included studies in the systematic review reported diagnostic accuracy data at different thresholds. Because of the low number of studies, meta-regression and subgroup analysis with pre-specified variables (i.e. blinding of the reference standard, distribution of diabetes type, percentage of depression cases in the sample and setting) could not be performed. Comparison between diabetes type 1 and type 2 could not be made because only one study included patients with diabetes type 1. Furthermore, the effect of the quality of the studies on the results could not be estimated, since the risk of bias in many studies was unclear in multiple domains. The diagnostic accuracy data could only be pooled at the usual thresholds. Since some symptoms of depression and diabetes overlap, the expectation was that higher thresholds would result in less false positives, and thus a higher specificity. Data about the NPV and PPV are of high value in the clinical setting. However, data about the NPV and PPV was not pooled, because these values are influenced by the prevalence of depression in the study populations.

No external ‘golden standard’ exists for diagnosing depression. A recent review by Petterson et al. suggests that the golden standard for diagnosing depression is the Longitudinal, Expert, All Data (LEAD) procedure in which all available data of a patient is taken into account as basis for diagnosis (i.e. information of family members, hospital records, psychological evaluation and laboratory results)[64]. However, a clinical interview is still the standard for diagnosing in clinical practice and was, therefore, incorporated as inclusion criterion. None of the included studies used the LEAD as reference standard.

The Grading of Recommendations, Assessment, Development and Evaluation (GRADE) approach could not be applied in the review. The GRADE-approach is a tool for “rating the quality of evidence and move from evidence to a recommendation”[65]. An essential component of formulating a recommendation is the patient-related outcomes of testing positive or negative on a depression questionnaire. These outcomes were not established in the included studies. A study into screening for depression in primary care found that “no trials have found better outcomes among patients who were screened than among patients who were not screened” because of low PPVs and small treatment effects[66]. It should be noticed that the prevalence of depression is higher in patients with diabetes than in the general population[2] which improves the PPV, and effective treatments are available for patients with diabetes[13, 14]. However, the number of false positives among patients with diabetes is still high.

Recent publications on diabetes and depression show the importance of subclinical depression [67] (i.e. clinically relevant depressive symptoms without fulfilling the criteria for major depressive disorder) and of diabetes-related emotional distress [68] (i.e. symptoms of depression and anxiety and disease specific related problems), as relevant constructs associated with increased depressive symptoms in people with diabetes or other comorbid chronic diseases. Depression plays an essential role in the course and prognosis of diabetes and other chronic diseases and must be recognized and treated in an early stage. Yet, we must be aware of the potential negative consequences of screening and diagnosing of patients at risk such as false positive screening results, high costs, additional burden and stigmatization.

### Conclusion

This review indicates that the CES-D (≥16) has the highest sensitivity, whereas the PHQ-9 (≥10) shows the highest specificity, yet confidence intervals were wide and overlapping.

#### Research implications

The results can aid future researchers to make better decisions in choosing questionnaires for the eligibility of participants in studies with patients with diabetes. The recommendation is to use the PHQ-9 (*≥*10) or the CES-D (*≥*16). The CES-D should be evaluated further, since best support was found regarding measurement properties for this questionnaire among patients with diabetes[27]. The PHQ-9 should be incorporated as well because this questionnaire yielded comparable results regarding sensitivity and specificity. Because other questionnaires (e.g. BDI, WHO-5 and HADS) are frequently used in clinical practice[1], these should be evaluated and tested more rigorously in the future. Future research could further estimate the diagnostic accuracy of depression questionnaires in the diabetes population. Focus should be on direct comparison of questionnaires to minimize the effect of bias; the use of higher thresholds to minimize the risk of overlap between symptoms of depression and diabetes; and trials to relate screening to use of screening questionnaires to patient-related outcomes in order to apply the GRADE-approach. The Standards for Reporting Diagnostic accuracy studies (STARD) guidelines help improve completeness of reporting[69].

#### Clinical implications

We suggest that the PHQ-9 (≥10) and the CES-D (≥16) are the most useful questionnaires for clinicians for the screening for depression among patients with diabetes. However, ultimately it is for clinicians to make an informed decision with a patient about the use of a depression questionnaire giving the aim, setting, time available and other relevant circumstances.

## Supporting information

S1 Table PRISMA-DTA checklist for abstract. (DOCX)

S2 Table PRISMA-DTA checklist. (DOCX)

S3 Table Search strategy and details of the removal of non-peer reviewed articles and duplicates. (DOCX)

S4 Table Answers on signaling questions of the QUADAS-2 per study. (DOCX)

S5 Table Characteristics of included questionnaires. (DOCX)

S6 Table Extracted data regarding diagnostic accuracy by questionnaire. (DOCX)

S1 Text Risk of bias assessment—signaling questions with interpretations. (DOCX)

S1 Fig SROC plots of the (A) CES-D (≥16), (B) PHQ-9(≥10) and (C) PHQ-9 algorithm.
(DOCX)
