# Supplementary material for: Diagnostic accuracy of depression questionnaires in adult patients with diabetes: A systematic review and meta-analysis
Source: PLoS One. 2019 Jun 20;14(6):e0218512. doi: 10.1371/journal.pone.0218512 (PMC6586329; doi:10.1371/journal.pone.0218512)
Supplement: S1 Table — (DOCX) [file pone.0218512.s001.docx]

**S1 Table. PRISMA-DTA Checklist for Abstract[1]**

| **Section/topic** | **#** | **PRISMA-DTA for Abstracts Checklist item** | **Reported on page #** |
| --- | --- | --- | --- |
| **TITLE and PURPOSE** | | |  |
| Title | 1 | Identify the report as a systematic review (+/- meta-analysis) of diagnostic test accuracy (DTA) studies. | 1 |
| Objectives | 2 | Indicate the research question, including components such as participants, index test, and target conditions. | 3 |
| **METHODS** | | |  |
| Eligibility criteria | 3 | Include study characteristics used as criteria for eligibility. | 3 |
| Information sources | 4 | List the key databases searched and the search dates. | 3 |
| Risk of bias & applicability | 5 | Indicate the methods of assessing risk of bias and applicability. | 3 |
| Synthesis of results | A1 | Indicate the methods for the data synthesis. | 3 |
| **RESULTS** | | |  |
| Included studies | 6 | Indicate the number and type of included studies and the participants and relevant characteristics of the studies (including the reference standard). | 3 |
| Synthesis of results | 7 | Include the results for the analysis of diagnostic accuracy, preferably indicating the number of studies and participants. Describe test accuracy including variability; if meta-analysis was done, include summary results and confidence intervals. | 3 |
| **DISCUSSION** | | |  |
| Strengths and limitations | 9 | Provide a brief summary of the strengths and limitations of the evidence | 3 |
| Interpretation | 10 | Provide a general interpretation of the results and the important implications. | 3 |
| **OTHER** | | |  |
| Funding | 11 | Indicate the primary source of funding for the review. | - |
| Registration | 12 | Provide the registration number and the registry name | 3 |

**References**

1. McInnes MF, Moher D, Thombs BD, McGrath TA, Bossuyt PM, Group atP-D. Preferred Reporting Items for a Systematic Review and Meta-analysis of Diagnostic Test Accuracy Studies: The PRISMA-DTA Statement. *JAMA* 2018; **319**(4): 388-96.
