# Supplementary material for: Diagnostic accuracy of depression questionnaires in adult patients with diabetes: A systematic review and meta-analysis
Source: PLoS One. 2019 Jun 20;14(6):e0218512. doi: 10.1371/journal.pone.0218512 (PMC6586329; doi:10.1371/journal.pone.0218512)
Supplement: S3 Table — (DOCX) [file pone.0218512.s003.docx]

S3 Table. Search strategy and details of the removal of non-peer reviewed articles and duplicates

| **Database** | **Date of search** | **Search terms** | **Selection options** | **Number of records** | **Number of unique articles** |
| --- | --- | --- | --- | --- | --- |
| PubMed | Beginning of literature - February 28^th^ 2018 | #1: (Depressive disorder[mh] OR depression[mh] OR (depress*[tiab] NOT medline[sb]))  #2: (Diabet*[tiab])  (#1 AND #2) | None used | 5,889 records   - 188 comments/ letters/ editorials/ book sections/ theses - 8 duplicates | 5,693 |
| Embase | Beginning of literature - February 28^th^ 2018 | #1: (Depression)  #2: (diabetes mellitus)  (#1 AND #2) | **Options:**   - Mapped to preferred term - Limit terms to index in article as ‘major focus’ - Explode   **Sources**:   - Embase   **Quick limits:**   - Humans - With abstract   **All years**  **Pub. Types**   - Articles - Articles in press - Review | 1,446 records   - 0 comments/ letters/ editorials/ book sections/ theses - 9 duplicates in Embase database - 1,114 duplicates removed after merging with PubMed database^a^ | 323 |
| PsycINFO | Beginning of literature - February 28^th^ 2018 | #1: (MM "Depression (Emotion)" OR TI depress*)  #2: (MM "Diabetes" OR MM "Diabetes Mellitus" OR MM "Type 2 Diabetes" OR TI diabetes)  (#1 AND #2) | None used | 884 records   - 139 comments/ letters/ editorials/ book sections/ theses - 0 duplicates in PsycINFO database - 664 duplicates removed after merging with PubMed and Embase database^b^ | 81 |
| **Total combined** | **Beginning of literature - February 28^th^ 2018** | **Not applicable** | **Not applicable** | **8,219 records**   - 327 non-peer reviewed articles - 1,795 duplicates | **6,097** |

^a^ records present in the PubMed database were removed according to the Bramer method^1^.

^b^ records present in the PubMed or Embase database were removed according to the Bramer method^1^

**References**

1. Bramer WM, Giustini D, de Jonge GB, Holland L, Bekhuis T. De-duplication of database search results for systematic reviews in EndNote. *J Med Libr Assoc* 2016; **104**(3): 240-3.
