# Supplementary material for: Diagnostic accuracy of depression questionnaires in adult patients with diabetes: A systematic review and meta-analysis
Source: PLoS One. 2019 Jun 20;14(6):e0218512. doi: 10.1371/journal.pone.0218512 (PMC6586329; doi:10.1371/journal.pone.0218512)
Supplement: S4 Table — (DOCX) [file pone.0218512.s004.docx]

**S4 Table. Answers on signaling questions of the QUADAS-2 per study**

| **Study** | **Patient selection** | | | | **Index test** | | | **Reference standard** | | | **Flow and timing** | | |
| --- | --- | --- | --- | --- | --- | --- | --- | --- | --- | --- | --- | --- | --- |
|  | **1.1** | **1.2** | **1.3** | **AC** | **2.1** | **2.2** | **AC** | **3.1** | **3.2** | **AC** | **4.1** | **4.2** | **4.3** |
| Ali (2013)[1] | Yes | Yes | No, only patients on oral anti-diabetic drugs, non-insulin users, maximum 5 years after diagnosis. | High, only patients on oral anti-diabetic drugs, non-insulin users, maximum 5 years after diagnosis. | Unclear | Yes | Low | Yes | Unclear | Low | Unclear | Yes | Unclear |
| Awata (2007)[2] | Yes | Yes | Yes | Low | Yes | Yes | Low | Yes | Yes | Low | Unclear | No, all screened positives + same number of screened negatives. Not stated if chosen randomly. | No because no random sample chosen from screened negatives and a lot of drop-out (129 🡪65). |
| Diaz-Rodriguez (2006)[3] | Yes | Yes | Yes | Low | Yes | Yes | Low | Yes | Unclear | Low | Yes, right after each other | Yes | Unclear, not mentioned. |
| Fisher (2007)[4] | Yes | Yes | No, patients with severe diabetes complications excluded | High, patients with severe diabetes complications excluded | Unclear | Yes | Low | Yes | Unclear | Low | Unclear | Yes | Yes, drop-out rate of 21.0%, but no differences in demographics and diabetes related factors. |
| Fisher (2016)[5] | Yes | Yes | Yes | Low | Unclear | Yes | Low | Yes | Unclear | Low | Unclear | Yes | Yes, drop-out rate of 20.9%, but no differences in demographics and diabetes related factors. |
| Hermanns (2006)[6] | Yes | Yes | Yes | Low | Yes | Yes | Low | Yes | Yes | Low | Unclear | No, only CIDI if patients were screened positive, SCA for identification false negatives. | No, 388 had complete data, only 376 in analysis. |

| **Study** | **Patient selection** | | | | **Index test** | | | **Reference standard** | | | **Flow and timing** | | |
| --- | --- | --- | --- | --- | --- | --- | --- | --- | --- | --- | --- | --- | --- |
|  | **1.1** | **1.2** | **1.3** | **AC** | **2.1** | **2.2** | **AC** | **3.1** | **3.2** | **AC** | **4.1** | **4.2** | **4.3** |
| Hsu (2014)[7] | Unclear | Yes | No, severe complications excluded | High, severe complications excluded | Unclear | Yes | Low | Unclear | Unclear | Low | Unclear | Unclear, unclear if participants underwent clinical assessment or assessment by psychiatrist as reference standard. | Yes, drop-out rate of approximately 0.5%. |
| Hyphantis (2015)[8] | Yes | Yes | Yes | Unclear, accident and emergency department | Yes | Yes | Low | Yes | Yes | Low | Unclear | Yes | Unclear, drop-out not specified for diabetes mellitus, only total population. |
| Janssen (2016)[9] | Yes | Yes | Yes | Low | Unclear | Yes | Low | Yes | Unclear | Low | Unclear, examination within three months: time between index test and reference standard or time between recruitment and assessment of depression. | Yes | Unclear, drop-out not specified for diabetes mellitus, only total population. |
| Khamseh (2011)[10] | Yes | Yes | No, no severe diabetes complications and no history of psychological disorders. | High, no severe diabetes complications and no history of psychological disorders. | Yes | Yes | Low | Yes | Yes | Low | Yes | Yes | Unclear |

| **Study** | **Patient selection** | | | | **Index test** | | | **Reference standard** | | | **Flow and timing** | | |
| --- | --- | --- | --- | --- | --- | --- | --- | --- | --- | --- | --- | --- | --- |
|  | **1.1** | **1.2** | **1.3** | **AC** | **2.1** | **2.2** | **AC** | **3.1** | **3.2** | **AC** | **4.1** | **4.2** | **4.3** |
| Krille (2008)[11] | Unclear | Yes | Unclear, not described | Low | Unclear | Yes | Low | Unclear | Unclear | Low | Unclear | Unclear. Whole sample received the reference standard. But it is mentioned that based on preliminary findings and at their own request the patients received the reference standard. | Unclear |
| Lamers (2008)[12] | Yes | Yes | No, age > 59 yrs, no diagnosis of major depression/ psychiatric conditions, current psychological/ psychiatric treatment | High, age > 59 yrs, no diagnosis of major depression/ psychiatric conditions, current psychological/ psychiatric treatment | Yes | Yes | Low | Yes | No | Low | Unclear | Yes, arbitrary sample of screened negatives | Unclear, dropout of total is given (422/5579) is given, not specified for diabetes mellitus. |
| Lustman (1997)[13] | Yes | Yes | Yes | High, only poor diabetes control | Unclear | Yes | Low | Yes | Yes | Low | Unclear | Yes | Unclear, not able to extract any data about recruitment/ response/proportion eligible unknown. |
| McHale (2008)[14] | Yes | Yes | Unclear, not described | Low | Yes | Yes | Low | Yes | Unclear | Low | Unclear | Yes | Yes, drop-out rate is 2%. |
| Stahl (2008)[15] | Yes | Yes | Yes | Low | Yes | Yes | Low | Yes | Unclear | Low | Yes | Yes | No, drop-out rate is 45.8%. |
| Sultan (2010)[16] | Yes | Yes | Yes | Low | Unclear | Yes | Low | Yes | Yes | Low | Yes | Yes | No, patients with dysthymia were excluded from analysis. |

| **Study** | **Patient selection** | | | | **Index test** | | | **Reference standard** | | | **Flow and timing** | | | |
| --- | --- | --- | --- | --- | --- | --- | --- | --- | --- | --- | --- | --- | --- | --- |
|  | **1.1** | **1.2** | **1.3** | **AC** | **2.1** | **2.2** | **AC** | **3.1** | **3.2** | **AC** | **4.1** | **4.2** | **4.3** | |
| Twist (2013)[17] | Yes | Yes | Yes | High, maximum duration of 6 months of type 2 diabetes. | Yes | Yes | Low | Yes | Unclear | Low | Yes | Yes, random sample is selected | No, not clear how many of screened negatives were excluded because of selection or refusal etc. 🡪 not all in the analysis. | |
| v. Steenbergen-Weijenburg (2010)[18] | Unclear | Yes | Yes | Low | Yes | Yes | Low | Yes | No | Low | Yes | Yes | No, high drop-out rates: screening process: 501/1278 excluded (did not fill in questionnaire) (39.2%); 395 eligible patients 🡪 198 dropped out (49.9%). | |
| Yoshida (2009)[19] | Yes | Yes | Yes | Low | Yes | Yes | Low | Unclear | Unclear | Low | Yes | No, participants who screened negative not randomly chosen + unclear if all psychiatrics did the same examinations or different | No, 197 recruited 🡪 129 for further evaluation, only 65 received reference standard. High drop-out in screened positives (34%), screened negatives not randomly chosen | |
| Zhang (2013)[20] | Yes | Yes | Yes | Low | Unclear | Yes | Low | Yes | Unclear | Low | Unclear | Yes, random sample is selected | Yes, all the patients who were randomly selected . | |
| Zhang (2015)[21] | Yes | Yes | Yes | Low | Unclear | Yes | Low | Yes | Unclear | Low | Unclear | Yes, random sample is selected | Yes, all the patients who were randomly selected . | |
| AC= applicability concern; CIDI= Composite International Diagnostic Interview; SCA= standardized clinical assessment. If no reason is given in the table, the reason is mentioned in the text about signaling questions at the page before the table | | | | | | | | | | | | | |  |

**References**

1. Ali N, Jyotsna VP, Kumar N, Mani K. Prevalence of depression among type 2 diabetes compared to healthy non diabetic controls. The Journal of the Association of Physicians of India. 2013;61(9):619-21. Epub 2014/04/30. PubMed PMID: 24772698.

2. Awata S, Bech P, Yoshida S, Hirai M, Suzuki S, Yamashita M, et al. Reliability and validity of the Japanese version of the World Health Organization-Five Well-Being Index in the context of detecting depression in diabetic patients. Psychiatry and clinical neurosciences. 2007;61(1):112-9. Epub 2007/01/24. doi: 10.1111/j.1440-1819.2007.01619.x. PubMed PMID: 17239048.

3. Diaz-Rodriguez G, Reyes-Morales H, Lopez-Caudana AE, Caraveo-Anduaga J, Atrian-Salazar ML. [Validation of a clinimetric scale for the diagnosis for depression in patients with diabetes mellitus type 2, in primary health care]. Revista de investigacion clinica; organo del Hospital de Enfermedades de la Nutricion. 2006;58(5):432-40. Epub 2007/04/06. PubMed PMID: 17408103.

4. Fisher L, Skaff MM, Mullan JT, Arean P, Mohr D, Masharani U, et al. Clinical Depression Versus Distress Among Patients With Type 2 Diabetes. Diabetes Care. 2007;30(3):542-8. doi: 10.2337/dc06-1614. PubMed PMID: WOS:000244941200014.

5. Fisher L, Hessler DM, Polonsky WH, Masharani U, Peters AL, Blumer I, et al. Prevalence of depression in Type 1 diabetes and the problem of over-diagnosis. Diabet Med. 2016;33(11):1590-7. Epub 2016/10/18. doi: 10.1111/dme.12973. PubMed PMID: 26433004.

6. Hermanns N, Kulzer B, Krichbaum M, Kubiak T, Haak T. How to screen for depression and emotional problems in patients with diabetes: comparison of screening characteristics of depression questionnaires, measurement of diabetes-specific emotional problems and standard clinical assessment. Diabetologia. 2006;49(3):469-77. doi: 10.1007/s00125-005-0094-2. PubMed PMID: 16432706.

7. Hsu LF, Kao CC, Wang MY, Chang CJ, Tsai PS. Psychometric testing of a Mandarin Chinese Version of the Clinically Useful Depression Outcome Scale for patients diagnosed with type 2 diabetes mellitus. International journal of nursing studies. 2014;51(12):1595-604. Epub 2014/06/22. doi: 10.1016/j.ijnurstu.2014.05.004. PubMed PMID: 24951085.

8. Hyphantis T, Kotsis K, Kroenke K, Paika V, Constantopoulos S, Drosos AA, et al. Lower PHQ-9 cutpoint accurately diagnosed depression in people with long-term conditions attending the Accident and Emergency Department. J Affect Disord. 2015;176:155-63. Epub 2015/02/28. doi: 10.1016/j.jad.2015.01.062. PubMed PMID: 25721612.

9. Janssen EP, Kohler S, Stehouwer CD, Schaper NC, Dagnelie PC, Sep SJ, et al. The Patient Health Questionnaire-9 as a Screening Tool for Depression in Individuals with Type 2 Diabetes Mellitus: The Maastricht Study. J Am Geriatr Soc. 2016;64(11):e201-e6. Epub 2016/10/27. doi: 10.1111/jgs.14388. PubMed PMID: 27783384.

10. Khamseh ME, Baradaran HR, Javanbakht A, Mirghorbani M, Yadollahi Z, Malek M. Comparison of the CES-D and PHQ-9 depression scales in people with type 2 diabetes in Tehran, Iran. BMC Psychiatry. 2011;11:61. Epub 2011/04/19. doi: 10.1186/1471-244x-11-61. PubMed PMID: 21496289; PubMed Central PMCID: PMCPMC3102614.

11. Krille S, Kulzer B, Reinecker H, Haak T, Hermanns N. Einflüsse von Psyche und Verhalten auf den Krankheitsverlauf (F54) bei Diabetes mellitus: Prävalenz und Screeningmethoden. Verhaltenstherapie & Verhaltensmedizin. 2008;29(4):323-35.

12. Lamers F, Jonkers CC, Bosma H, Penninx BW, Knottnerus JA, van Eijk JT. Summed score of the Patient Health Questionnaire-9 was a reliable and valid method for depression screening in chronically ill elderly patients. J Clin Epidemiol. 2008;61(7):679-87. Epub 2008/06/10. doi: 10.1016/j.jclinepi.2007.07.018. PubMed PMID: 18538262.

13. Lustman PJ, Clouse RE, Griffith LS, Carney RM, Freedland KE. Screening for depression in diabetes using the Beck Depression Inventory. Psychosomatic medicine. 1997;59(1):24-31. Epub 1997/01/01. PubMed PMID: 9021863.

14. McHale M, Hendrikz J, Dann F, Kenardy J. Screening for depression in patients with diabetes mellitus. Psychosomatic medicine. 2008;70(8):869-74. Epub 2008/10/10. doi: 10.1097/PSY.0b013e318186dea9. PubMed PMID: 18842744.

15. Stahl D, Sum CF, Lum SS, Liow PH, Chan YH, Verma S, et al. Screening for depressive symptoms: validation of the center for epidemiologic studies depression scale (CES-D) in a multiethnic group of patients with diabetes in Singapore. Diabetes Care. 2008;31(6):1118-9. Epub 2008/03/14. doi: 10.2337/dc07-2019. PubMed PMID: 18337303.

16. Sultan S, Luminet O, Hartemann A. Cognitive and anxiety symptoms in screening for clinical depression in diabetes: a systematic examination of diagnostic performances of the HADS and BDI-SF. J Affect Disord. 2010;123:332-6. Epub 2009/10/29. doi: 10.1016/j.jad.2009.09.022. PubMed PMID: 19861228.

17. Twist K, Stahl D, Amiel SA, Thomas S, Winkley K, Ismail K. Comparison of depressive symptoms in type 2 diabetes using a two-stage survey design. Psychosomatic medicine. 2013;75(8):791-7. Epub 2013/08/08. doi: 10.1097/PSY.0b013e3182a2b108. PubMed PMID: 23922402.

18. van Steenbergen-Weijenburg KM, de Vroege L, Ploeger RR, Brals JW, Vloedbeld MG, Veneman TF, et al. Validation of the PHQ-9 as a screening instrument for depression in diabetes patients in specialized outpatient clinics. BMC Health Serv Res. 2010;10(1):235. doi: 10.1186/1472-6963-10-235. PubMed PMID: 20704720; PubMed Central PMCID: PMCPMC2927590.

19. Yoshida S, Hirai M, Suzuki S, Awata S, Oka Y. Neuropathy is associated with depression independently of health-related quality of life in Japanese patients with diabetes. Psychiatry and clinical neurosciences. 2009;63(1):65-72. Epub 2008/12/11. doi: 10.1111/j.1440-1819.2008.01889.x. PubMed PMID: 19067994.

20. Zhang Y, Ting R, Lam M, Lam J, Nan H, Yeung R, et al. Measuring depressive symptoms using the Patient Health Questionnaire-9 in Hong Kong Chinese subjects with type 2 diabetes. J Affect Disord. 2013;151(2):660-6. Epub 2013/08/14. doi: 10.1016/j.jad.2013.07.014. PubMed PMID: 23938133.

21. Zhang Y, Ting RZ, Lam MH, Lam SP, Yeung RO, Nan H, et al. Measuring depression with CES-D in Chinese patients with type 2 diabetes: the validity and its comparison to PHQ-9. BMC Psychiatry. 2015;15:198. Epub 2015/08/19. doi: 10.1186/s12888-015-0580-0. PubMed PMID: 26281832; PubMed Central PMCID: PMCPMC4538746.
