# Supplementary material for: Diagnostic accuracy of depression questionnaires in adult patients with diabetes: A systematic review and meta-analysis
Source: PLoS One. 2019 Jun 20;14(6):e0218512. doi: 10.1371/journal.pone.0218512 (PMC6586329; doi:10.1371/journal.pone.0218512)
Supplement: S5 Table — (DOCX) [file pone.0218512.s005.docx]

S5 Table. Characteristics of included questionnaires

| **Questionnaire** | **Construct aimed to be measured** | **Target population** | **#items** | **Score range** | | **Usual thresholds for depression** | **Administration time** | **Recall period** |
| --- | --- | --- | --- | --- | --- | --- | --- | --- |
|  |  |  |  | **item** | **total** |  |  |  |
| **BDI** ^1^ | Intensity of depressive symptoms | General population | 21 | 0-3 | 0-63 | <10: none or minimal | 5-10 minutes | 1 week |
|  |  |  |  |  |  | 10-18: mild to moderate |  |  |
|  |  |  |  |  |  | 19-29: moderate/severe |  |  |
|  |  |  |  |  |  | ≥30: severe |  |  |
| **BDI-SF** ^2^ | Intensity of depressive symptoms | General population | 13 | 0-3 | 0-39 | ≥8^a^ | 3-7 minutes | 1 week |
|  |  |  | BDIcog (8) |  | 0-24 | ≥3^a^ |  |  |
|  |  |  | BDIsom(5) |  | 0-15 | ≥4^a^ |  |  |
| **CES-D** ^3^ | Level of depressive symptomatology | General population | 20 | 0-3 | 0-60 | ≥16 | 5-10 minutes | 1 week |
| **CSDD** ^4^ | Depression | Diabetes patients | 13 | 0-1 | 0-13 | ≥6^a^ | Not specified | 2 weeks |
| **CUDOS** ^5^ | Depression | General population | 18 | 0-4 | 0-72 | 0-10: no depression | 3 minutes | 1 week |
|  |  |  |  |  |  | 11-20: minimal |  |  |
|  |  |  |  |  |  | 21-30: mild |  |  |
|  |  |  |  |  |  | 31-45: moderate |  |  |
|  |  |  |  |  |  | >45: severe |  |  |
| **DMI-10** ^6^ | Cognitive aspects of depression | Medically ill patients | 10 | 0-3 | 0-30 | ≥ 9 | Not specified | 2-3 days |
| **HADS-D** ^7^ | Depression | Hospital outpatient | 7 | 0-3 | 0-21 | 8-10: mild | 2-5 minutes | 1 week |
|  |  |  |  |  |  | ≥11: moderate/severe |  |  |
| **PHQ-9 ^8^** | Symptoms of major depressive disorder | Primary care patients | 9 | 0-3 | 0-27 | 0-4: no depression | Within minutes | 2 weeks |
|  |  |  | PHQ-8(8)^b^ |  | 0-24 | 5-9: minimal |  |  |
|  |  |  |  |  |  | 10-14: mild |  |  |
|  |  |  |  |  |  | 15-19: moderate |  |  |
|  |  |  |  |  |  | ≥ 20: severe |  |  |
| **SCAD** ^9^ | Major depressive disorder | Medically ill patients | 6 | 0-1 | 0-6 | ≥ 3 | Several minutes | 1 week |
| **SDS** ^10^ | Depressive disorders | Hospitalized patients and hospital outpatients | 20 | 1-4 | 25-100^c^ | <50: no depression^c^ | Not specified | Not specified |
|  |  |  |  |  |  | 50-59: mild |  |  |
|  |  |  |  |  |  | 60-69: moderate |  |  |
|  |  |  |  |  |  | ≥70: severe |  |  |
| **WHO-5** ^11^ | Emotional well-being (later: depression) | General population | 5 | 0-5 | 0-100^d^ | ≤50 clinical depression^d^ | <5 minutes | 2 weeks |
|  |  |  |  |  |  | ≤28 major depression^d^ |  |  |
| BDI= Beck Depression Inventory; BDI-SF= Beck Depression Inventory-Short Form (divided in subscales: cognitive (BDIcog) and somatic (BDIsom)); CES-D= Centre for Epidemiological Studies Depression Scale; CSDD= Clinimetric Scale for the Diagnosis of Depression; CUDOS= Clinically Useful Depression Outcome Scale; DMI-10= Depression in the Medically Ill; HADS-D= Hospital Anxiety and Depression Scale - Depression; PHQ-8= Patient Health Questionnaire 8-item version; PHQ-9= Patient Health Questionnaire 9-item version; SCAD= Silverstone Concise Assessment for Depression; SDS= Zung Self rating Depression Scale;WHO-5= World Health Organization-Five Well-Being Index.  ^a^ Threshold based on the included study  ^b^ PHQ-8 corresponds with PHQ-9 without question about suicidal thoughts  ^c^ Sum score of total SDS is multiplied by 1.25, included study did not do this: threshold ≥40 (range 20-80) ^12^  ^d^ Sum score of total WHO-5 is multiplied by 4, included studies did not do this: threshold ≤13 (range 0-25)^13,14^ | | | | | | | | |

**References**

1. Beck AT, Steer RA, Brown GK. Manual for the Beck Depression Inventory-II. 2nd ed. San Antonio, Tex.: Psychological Corporation; 1996.

2. Beck AT, Beck RW. Screening Depressed Patients in Family Practice. *Postgraduate Medicine* 1972; **52**(6): 81-5.

3. Lenore Sawyer R. The CES-D Scale: A Self-Report Depression Scale for Research in the General Population. *Applied Psychological Measurement* 1977; **1**(3): 385-401.

4. Diaz-Rodriguez G, Reyes-Morales H, Lopez-Caudana AE, Caraveo-Anduaga J, Atrian-Salazar ML. [Validation of a clinimetric scale for the diagnosis for depression in patients with diabetes mellitus type 2, in primary health care]. *Revista de investigacion clinica; organo del Hospital de Enfermedades de la Nutricion* 2006; **58**(5): 432-40.

5. Zimmerman M, Chelminski I, McGlinchey JB, Posternak MA. A clinically useful depression outcome scale. *Compr Psychiatry* 2008; **49**(2): 131-40.

6. Parker G, Hilton T, Bains J, Hadzi-Pavlovic D. Cognitive-based measures screening for depression in the medically ill: the DMI-10 and the DMI-18. *Acta Psychiatr Scand* 2002; **105**(6): 419-26.

7. Zigmond AS, Snaith RP. The Hospital Anxiety and Depression Scale. *Acta Psychiatr Scand* 1983; **67**(6): 361-70.

8. Kroenke K, Spitzer RL. The PHQ-9: A New Depression Diagnostic and Severity Measure. *Psychiatric Annals* 2002; **32**(9): 509-15.

9. Silverstone PH. Concise assessment for depression (CAD): A brief screening approach to depression in the medically ill. *J Psychosom Res* 1996; **41**(2): 161-70.

10. Zung WWK. A Self-Rating Depression Scale. *Arch Gen Psychiat* 1965; **12**(1): 63-70.

11. Topp CW, Østergaard SD, Søndergaard S, Bech P. The WHO-5 Well-Being Index: A Systematic Review of the Literature. *Psychother Psychosom* 2015; **84**(3): 167-76.

12. Yoshida S, Hirai M, Suzuki S, Awata S, Oka Y. Neuropathy is associated with depression independently of health-related quality of life in Japanese patients with diabetes. *Psychiatry and clinical neurosciences* 2009; **63**(1): 65-72.

13. Awata S, Bech P, Yoshida S, et al. Reliability and validity of the Japanese version of the World Health Organization-Five Well-Being Index in the context of detecting depression in diabetic patients. *Psychiatry and clinical neurosciences* 2007; **61**(1): 112-9.

14. Krille S, Kulzer B, Reinecker H, Haak T, Hermanns N. Einflüsse von Psyche und Verhalten auf den Krankheitsverlauf (F54) bei Diabetes mellitus: Prävalenz und Screeningmethoden. *Verhaltenstherapie & Verhaltensmedizin* 2008; **29**(4): 323-35.
