# Supplementary material for: Diagnostic accuracy of depression questionnaires in adult patients with diabetes: A systematic review and meta-analysis
Source: PLoS One. 2019 Jun 20;14(6):e0218512. doi: 10.1371/journal.pone.0218512 (PMC6586329; doi:10.1371/journal.pone.0218512)
Supplement: S6 Table — (DOCX) [file pone.0218512.s006.docx]

S6 Table. Extracted data regarding diagnostic accuracy by questionnaire

| Questionnaire | Study | Version of index test (threshold) ^a^ | Used reference | Diagnostic accuracy | | | | |
| --- | --- | --- | --- | --- | --- | --- | --- | --- |
|  |  |  |  | AUC  (95%CI) or ±SE | Sensitivity (%) (95%CI) | Specificity (%) (95%CI) | PPV (%)  (95%CI) | NPV (%)  (95%CI) |
| BDI | Ali (2013)[1] | Full BDI (16) | MINI | .91 (n.r.) | 100 (n.r.) | 71 (n.r.) | n.r | n.r. |
|  |  | **Full BDI (21)** |  |  | **85 (n.r.)** | **80 (n.r.)** | **n.r.** | **n.r.** |
|  | Hermanns (2006)[2] | **Full BDI (7)** | CIDI or SCA | .85 ±0.02 | **n.r.** | **n.r.** | **n.r.** | **n.r.** |
|  |  | Full BDI (10) |  |  | 87 (83-90) | 81 (78-85) | 43 (38- 48) | 97 (96-99) |
|  | Lustman (1997)[3] | BDIcog | DIS-revised | .93 ±0.02 | n.r. | n.r. | n.r. | n.r. |
|  |  | BDIsom |  | .82 ±0.03 |  |  |  |  |
|  |  | Full BDI (8)  Full BDI (10)  Full BDI (12)  **Full BDI (13)**  Full BDI (14)  Full BDI (16) |  | .94 ±0.02 | 99 (n.r.) | 52 (n.r.) | n.r. | n.r. |
|  |  |  |  |  | 98 (n.r.) | 70 (n.r.) |  |  |
|  |  |  |  |  | 90 (n.r.) | 84 (n.r.) |  |  |
|  |  |  |  |  | **85 (n.r.)** | **88 (n.r.)** |  |  |
|  |  |  |  |  | 82 (n.r.) | 89 (n.r.) |  |  |
|  |  |  |  |  | 73 (n.r.) | 93 (n.r.) |  |  |
|  | Sultan (2010)[4] ^b^ | BDI-SF (4) | MINI | .90 (.81-.94) | 100 (100-100) | 47 (41-53) | 18 (12-24) | 100 (100-100) |
|  |  | **BDI-SF (8)** |  |  | **77 (62-92)** | **81 (76-86)** | **32 (22-43)** | **97 (94-99)** |
|  |  | BDI-SF (16) |  |  | 37 (19-54) | 98 (97-100) | 73 (51-96) | 93 (90-96) |
|  |  | **BDI-SFcog (4)** |  | .90 (.82-.94) | **83 (70-97)** | **80 (75-85)** | **33 (22-43)** | **98 (96-100)** |
|  |  | **BDI-SFsom (4)** |  | n.r. | **73 (58-89)** | **68 (62-74)** | **21 (13-29)** | **96 (93-99)** |
| CES-D | Fisher (2007)[5] | Full CES-D (16) | CIDI (Dx1) | n.r. | 78 (n.r.) | n.r. | n.r. | n.r. |
|  | Hermanns (2006)[2] | **Full CES-D (14)** | CIDI or SCA | .80 ±0.02 | **n.r.** | **n.r.** | **n.r.** | **n.r.** |
|  |  | Full CES-D (23) |  |  | 79 (75-83) | 89 (86-92) | 54 (49-59) | 96 (94-98) |
|  | Khamseh (2011)[6] | Full CES-D (16) | SCID | .86 ±0.03 | 90 (81-95) | 60 (50-69) | n.r. | n.r. |
|  |  | Full CES-D (22) |  |  | 83 (72-90) | 77 (68-85) |  |  |
|  |  | **Full CES-D (23)** |  |  | **79 (68-87)** | **77 (68-85)** |  |  |
|  | McHale (2008)[7] | Full CES-D (13) | CIDI-SF | .81 (.74-.88) | 71 (n.r.) | 71 (n.r.) | 51 (n.r.) | 85 (n.r.) |
|  |  | Full CES-D (16) |  |  | 61 (n.r.) | 81 (n.r.) | 58 (n.r.) | 83 (n.r.) |
|  | Stahl (2008)[8] ^c^ | Full CES-D C (16)^c^ | SCAN | .82 (n.r.) | 97 (n.r.) | 68 (n.r.) | 35 (n.r.) | 99 (n.r.) |
|  |  | Full CES-D M (16)^c^ |  | .64 (n.r.) | 67 (n.r.) | 61 (n.r.) | 24 (n.r.) | 91 (n.r.) |
|  |  | Full CES-D I (16)^c^ |  | .82 (n.r.) | 100 (n.r.) | 65 (n.r.) | 56 (n.r.) | 100 (n.r.) |
|  | Zhang (2015)[9] | Full CES-D (16) | MINI | .85 (.77-.92) | 91 (n.r.) | 61 (n.r.) | 42 (n.r.) | 96 (n.r.) |
|  |  | Full CES-D (17) |  |  | 87 (n.r.) | 65 (n.r.) | 44 (n.r.) | 94 (n.r.) |
|  |  | Full CES-D (18) |  |  | 78 (n.r.) | 68 (n.r.) | 43 (n.r.) | 91 (n.r.) |
|  |  | Full CES-D (19) |  |  | 78 (n.r.) | 72 (n.r.) | 46 (n.r.) | 91 (n.r.) |
|  |  | Full CES-D (20) |  |  | 78 (n.r.) | 72 (n.r.) | 46 (n.r.) | 91 (n.r.) |
|  |  | **Full CES-D (21)** |  |  | **78 (n.r.)** | **74 (n.r.)** | **49 (n.r.)** | **92 (n.r.)** |
|  |  | Full CES-D (22) |  |  | 74 (n.r.) | 77 (n.r.) | 50 (n.r.) | 91 (n.r.) |
|  |  | 16-item CES-D (13) |  | .85 (.77-.94) | 83 (n.r.) | 73 (n.r.) | 49 (n.r.) | 93 (n.r.) |
|  |  | **16-item CES-D (14)** |  |  | **83 (n.r.)** | **74 (n.r.)** | **50 (n.r.)** | **93 (n.r.)** |
|  |  | 16-item CES-D (15) |  |  | 74 (n.r.) | 78 (n.r.) | 52 (n.r.) | 91 (n.r.) |

| **Questionnaire** | **Study** | **Version of index test (threshold) ^a^** | **Used reference** | **Diagnostic accuracy** | | | | |
| --- | --- | --- | --- | --- | --- | --- | --- | --- |
|  |  |  |  | **AUC  (95%CI) or ±SE** | **Sensitivity (%) (95%CI)** | **Specificity (%) (95%CI)** | PPV (%)  **(95%CI)** | **NPV (%)  (95%CI)** |
| CSDD | Diaz-Rodriguez (2006)[10] | Full CSDD (11) | CIDI | .94 (n.r.) | 89 (n.r.) | 99 (n.r.) | 73 (n.r.) | 100 (n.r.) |
| CUDOS | Hsu (2014)[11] | **CUDOS (19/20)** | DSM-IV (n.s.) | **.84 (.77-.90)** | **78 (n.r.)** | **76 (n.r.)** | **n.r.** | **n.r.** |
| DMI-10 | McHale (2008)[7] | Full DMI-10 (6) | CIDI | .77 (.68-.86) | 71 (n.r.) | 72 (n.r.) | 52 (n.r.) | 85 (n.r.) |
|  |  | Full DMI-10 (9) |  |  | 57 (n.r.) | 86 (n.r.) | 64 (n.r.) | 82 (n.r.) |
| HADS-D | McHale (2008)[7] | HADS-D (5)  HADS-D (11) | CIDI | .72 (.62-.81) | 64 (n.r.)  23 (n.r.) | 64 (n.r.)  98 (n.r.) | 43 (n.r.)  83 (n.r.) | 81 (n.r.)  75 (n.r.) |
|  |  |  |  |  |  |  |  |  |
|  | Sultan (2010)[4] ^b^ | **HADS-D (7)** | MINI | .80 (.71-.89) | **73 (58-89)** | **79 (74-84)** | **29 (19-39)** | **96 (94-99)** |
|  |  | HADS-D (8) |  |  | 53 (35-71) | 86 (81-90) | 30 (18-43) | 94 (91-97) |
|  |  | HADS-D (11) |  |  | 27 (11-42) | 97 (95-99) | 53 (28-79) | 92 (89-95) |
| PHQ-8 | Fisher (2016)[12] | PHQ-8 (10) | SCID | n.r. | 92 (n.r.) | 91 (n.r.) | 29 (n.r.) | 99 (n.r.) |
|  |  | PHQ-8 (12) |  |  | 69 (n.r.) | 95 (n.r.) | 34 (n.r.) | 99 (n.r.) |
|  |  | PHQ-8 (15) |  |  | 46 (n.r.) | 98 (n.r.) | 43 (n.r.) | 98 (n.r.) |
|  |  | PHQ-8 (algorithm) |  |  | 61 (n.r.) | 97 (n.r.) | 47 (n.r.) | 99 (n.r.) |
| PHQ-9 | Hyphantis (2015)[13] | **Full PHQ-9 (6)** | MINI | **.96 (n.r.)** | **98 (88-100)** | **85 (78-90)** | **n.r.** | **n.r.** |
|  | Janssen (2016)[14] | Full PHQ-9 (3) | MINI | .87 (n.r) | 98 (n.r.) | 61 (n.r.) | 12 (n.r.) | 100 (n.r.) |
|  |  | **Full PHQ-9 (5)** |  |  | **92 (n.r.)** | **70 (n.r.)** | **15 (n.r.)** | **99 (n.r.)** |
|  |  | Full PHQ-9 (7) |  |  | 81 (n.r.) | 88 (n.r.) | 27 (n.r.) | 99 (n.r.) |
|  |  | Full PHQ-9 (10) |  |  | 43 (n.r.) | 96 (n.r.) | 46 (n.r.) | 96 (n.r.) |
|  | Khamseh (2011)[6] | Full PHQ-9 (10) | SCID | .83 ±0.30 | 84 (73-91) | 66 (56-75) | n.r. | n.r. |
|  |  | **Full PHQ-9 (13)** |  |  | **74 (63-83)** | **76 (67-84)** |  |  |
|  |  | Full PHQ-9 (algorithm) |  | not applicable | 78 (67-86) | 75 (66-83) |  |  |
|  | Lamers (2008)[15] | **Full PHQ-9 (7)** | MINI | n.r. | **91 (n.r.)** | **81 (n.r.)** | **n.r.** | **n.r.** |
|  |  | Full PHQ-9 (algorithm) |  | not applicable | 35 (31-39) | 97 (96-97) | 69 (62-74) | 87 (86-89) |
|  | Twist (2013)[16] | Full PHQ-9 (10) | SCAN 2.1 | .92 (.89-.95) | 95 (n.r.) | 70 (n.r.) | 24 (n.r.) | 99 (n.r.) |
|  |  | Full PHQ-9 (11) |  |  | 89 (n.r.) | 76 (n.r.) | 27 (n.r.) | 99 (n.r.) |
|  |  | **Full PHQ-9 (12)** |  |  | **87 (n.r.)** | **80 (n.r.)** | **30 (n.r.)** | **98 (n.r.)** |
|  |  | Full PHQ-9 (13) |  |  | 81 (n.r.) | 84 (n.r.) | 33 (n.r.) | 98 (n.r.) |
|  |  | Full PHQ-9 (14) |  |  | 77 (n.r.) | 87 (n.r.) | 38 (n.r.) | 98 (n.r.) |
|  | v. Steenbergen- Weijenburg (2010)[17] | Full PHQ-9 (8) | MINI | .77 (.69-.84) | 92 (n.r.) | 59 (n.r.) | 34 (n.r.)^d^ | 97 (n.r.)^d^ |
|  |  | Full PHQ-9 (9) |  |  | 92 (n.r.) | 62 (n.r.) | 36 (n.r.)^d^ | 97 (n.r.)^d^ |
|  |  | Full PHQ-9 (10) |  |  | 92 (n.r.) | 64 (n.r.) | 37 (n.r.)^d^ | 97 (n.r.)^d^ |
|  |  | Full PHQ-9 (11) |  |  | 81 (n.r.) | 74 (n.r.) | 37 (n.r.)^d^ | 94 (n.r.)^d^ |
|  |  | **Full PHQ-9 (12)** |  |  | **76 (n.r.)** | **80 (n.r.)** | **47 (n.r.)^d^** | **93 (n.r.)^d^** |
|  |  | Full PHQ-9 (algor 0-27)  Full PHQ-9 (algor >10)^e^ |  | not applicable | 58 (n.r.)  64 (n.r.) | 87 (n.r.)  64 (n.r.) | 50 (n.r.)  50 (n.r.) | 90 (n.r.)  76 (n.r.) |

| **Questionnaire** | **Study** | **Version of index test (threshold) ^a^** | **Used reference** | **Diagnostic accuracy** | | | | | |
| --- | --- | --- | --- | --- | --- | --- | --- | --- | --- |
|  |  |  |  | **AUC  (95%CI) or ±SE** | **Sensitivity (%) (95%CI)** | **Specificity (%) (95%CI)** | PPV (%)  **(95%CI)** | **NPV (%)  (95%CI)** | |
| PHQ-9 (continued) | Zhang (2013)[18] | Full PHQ-9 (6) | MINI | .85 (.76- .94) | 87 (n.r.) | 70 (n.r.) | 37 (n.r.) | 96 (n.r.) | |
|  |  | **Full PHQ-9 (7)** |  |  | **83 (n.r.)** | **74 (n.r.)** | **39 (n.r.)** | **95 (n.r.)** | |
|  |  | Full PHQ-9 (8) |  |  | 74 (n.r.) | 78 (n.r.) | 40 (n.r.) | 94 (n.r.) | |
|  |  | Full PHQ-9 (9) |  |  | 61 (n.r.) | 84 (n.r.) | 44 (n.r.) | 91 (n.r.) | |
|  |  | Full PHQ-9 (10) |  |  | 57 (n.r.) | 84 (n.r.) | 42 (n.r.) | 91 (n.r.) | |
|  |  | Full PHQ-9 algorithm |  | not applicable | 39 (n.r.) | 96 (n.r.) | 75 (n.r.) | 84 (n.r.) | |
| SCAD | McHale (2008)[7] | Full SCAD (1) | CIDI | .73 (.65- .81) | 80 (n.r.) | 64 (n.r.) | 48 (n.r.) | 88 (n.r.) | |
|  |  | Full SCAD (3) |  |  | 64 (n.r.) | 72 (n.r.) | 49 (n.r.) | 82 (n.r.) | |
| WHO-5 | Awata (2007)[19] | **Full WHO-5 (13)** | **SCID-I** | **.90 (.81-.98)** | **100 (n.r.)** | **74 (n.r.)** | **32 (n.r.)** | **100 (n.r.)** | |
|  | Krille (2008)[20] | Full WHO-5 (7) | Clinical interview  (n.s.) | .81 (.75-.87) | 44 (n.r.) | 84 (n.r) | 21 (n.r.) | 94 (n.r.) | |
|  |  | **Full WHO-5 (11)** |  |  | **78 (n.r.)** | **70 (n.r.)** | **n.r.** | **n.r.** | |
|  |  | Full WHO-5 (13) |  |  | 100 (n.r.) | 59 (n.r.) | 20 (n.r.) | 100 (n.r.) | |
| SDS | Yoshida (2009)[21] | Full SDS (40) | DSM-IV (n.s.) | n.r. | 100 (n.r.) | 59 (n.r.) | n.r. | n.r. | |
| 95%CI= 95% confidence interval; AUC= area under the curve; BDI= Beck Depression Inventory; BDI-SF= Beck Depression Inventory-Short Form; BDI-SFcog= cognitive subset of items of BDI-SF; BDI-SFsom= somatic subset of items of BDI-SF; BDIcog= cognitive subset of items of BDI; BDIsom= somatic subset of items of BDI; CES-D= Centre for Epidemiological Studies Depression Scale; CIDI= Composite International Diagnostic Interview; CIDI (Dx1)= Composite International Diagnostic Interview Depression within the last month; CIDI-SF= Composite International Diagnostic Interview- Short Form; CSDD= Clinimetric Scale for the Diagnosis of Depression; CUDOS= Clinically Useful Depression Outcome Scale; DIS-revised= National Institute of Mental Health Diagnostic Interview Schedule - Version IIIR; DMI-10= Depression in the Medically Ill; DSM-IV= Diagnostic and Statistical Manual of Mental Disorders - Version IV;HADS-D= Hospital Anxiety and Depression Scale - Depression; MINI= Mini International Neuropsychiatric Interview; NPV= negative predictive value; n.r.= not reported; n.s.= not specified; PHQ-8= Patient Health Questionnaire 8-item version; PHQ-9= Patient Health Questionnaire 9-item version; PPV= positive predictive value; SCA= standardized clinical assessment; SCAD= Silverstone Concise Assessment for Depression; SCAN (2.1)= Schedule for Clinical Assessment in Neuropsychiatry; SCID= Structured Clinical Interview for DSM Disorders; SCID-I= Structured Clinical Interview for DSM Disorders- Axis I; SDS= Zung Self rating Depression Scale; SE= standard error; WHO-5= World Health Organization-Five Well-Being Index.  ^a^ Found optimal threshold in bold. When no threshold is in bold, the optimal was not given in the article.  ^b^ Patients with dysthymia were excluded from the analysis, resulting in a sample size of 286 patients.  ^c^ Diagnostic accuracy data was split up per population (i.e. Chinese (C), Malay (M) and Indian(I))  ^d^ Data of NPV and PPV of thresholds 8 to 12 was exchanged, based on abstract and two-by-two-tables derived from sensitivity and specificity data.  ^e^ PHQ-9 (algorithm>10): diagnostic accuracy data of the PHQ-9 (algorithm) among the participants who scored above 10 on the PHQ-9 (n=91) | | | | | | | | |  |

**References**

1. Ali N, Jyotsna VP, Kumar N, Mani K. Prevalence of depression among type 2 diabetes compared to healthy non diabetic controls. The Journal of the Association of Physicians of India. 2013;61(9):619-21. Epub 2014/04/30. PubMed PMID: 24772698.

2. Hermanns N, Kulzer B, Krichbaum M, Kubiak T, Haak T. How to screen for depression and emotional problems in patients with diabetes: comparison of screening characteristics of depression questionnaires, measurement of diabetes-specific emotional problems and standard clinical assessment. Diabetologia. 2006;49(3):469-77. doi: 10.1007/s00125-005-0094-2. PubMed PMID: 16432706.

3. Lustman PJ, Clouse RE, Griffith LS, Carney RM, Freedland KE. Screening for depression in diabetes using the Beck Depression Inventory. Psychosomatic medicine. 1997;59(1):24-31. Epub 1997/01/01. PubMed PMID: 9021863.

4. Sultan S, Luminet O, Hartemann A. Cognitive and anxiety symptoms in screening for clinical depression in diabetes: a systematic examination of diagnostic performances of the HADS and BDI-SF. J Affect Disord. 2010;123:332-6. Epub 2009/10/29. doi: 10.1016/j.jad.2009.09.022. PubMed PMID: 19861228.

5. Fisher L, Skaff MM, Mullan JT, Arean P, Mohr D, Masharani U, et al. Clinical Depression Versus Distress Among Patients With Type 2 Diabetes. Diabetes Care. 2007;30(3):542-8. doi: 10.2337/dc06-1614. PubMed PMID: WOS:000244941200014.

6. Khamseh ME, Baradaran HR, Javanbakht A, Mirghorbani M, Yadollahi Z, Malek M. Comparison of the CES-D and PHQ-9 depression scales in people with type 2 diabetes in Tehran, Iran. BMC Psychiatry. 2011;11:61. Epub 2011/04/19. doi: 10.1186/1471-244x-11-61. PubMed PMID: 21496289; PubMed Central PMCID: PMCPMC3102614.

7. McHale M, Hendrikz J, Dann F, Kenardy J. Screening for depression in patients with diabetes mellitus. Psychosomatic medicine. 2008;70(8):869-74. Epub 2008/10/10. doi: 10.1097/PSY.0b013e318186dea9. PubMed PMID: 18842744.

8. Stahl D, Sum CF, Lum SS, Liow PH, Chan YH, Verma S, et al. Screening for depressive symptoms: validation of the center for epidemiologic studies depression scale (CES-D) in a multiethnic group of patients with diabetes in Singapore. Diabetes Care. 2008;31(6):1118-9. Epub 2008/03/14. doi: 10.2337/dc07-2019. PubMed PMID: 18337303.

9. Zhang Y, Ting RZ, Lam MH, Lam SP, Yeung RO, Nan H, et al. Measuring depression with CES-D in Chinese patients with type 2 diabetes: the validity and its comparison to PHQ-9. BMC Psychiatry. 2015;15:198. Epub 2015/08/19. doi: 10.1186/s12888-015-0580-0. PubMed PMID: 26281832; PubMed Central PMCID: PMCPMC4538746.

10. Diaz-Rodriguez G, Reyes-Morales H, Lopez-Caudana AE, Caraveo-Anduaga J, Atrian-Salazar ML. [Validation of a clinimetric scale for the diagnosis for depression in patients with diabetes mellitus type 2, in primary health care]. Revista de investigacion clinica; organo del Hospital de Enfermedades de la Nutricion. 2006;58(5):432-40. Epub 2007/04/06. PubMed PMID: 17408103.

11. Hsu LF, Kao CC, Wang MY, Chang CJ, Tsai PS. Psychometric testing of a Mandarin Chinese Version of the Clinically Useful Depression Outcome Scale for patients diagnosed with type 2 diabetes mellitus. International journal of nursing studies. 2014;51(12):1595-604. Epub 2014/06/22. doi: 10.1016/j.ijnurstu.2014.05.004. PubMed PMID: 24951085.

12. Fisher L, Hessler DM, Polonsky WH, Masharani U, Peters AL, Blumer I, et al. Prevalence of depression in Type 1 diabetes and the problem of over-diagnosis. Diabet Med. 2016;33(11):1590-7. Epub 2016/10/18. doi: 10.1111/dme.12973. PubMed PMID: 26433004.

13. Hyphantis T, Kotsis K, Kroenke K, Paika V, Constantopoulos S, Drosos AA, et al. Lower PHQ-9 cutpoint accurately diagnosed depression in people with long-term conditions attending the Accident and Emergency Department. J Affect Disord. 2015;176:155-63. Epub 2015/02/28. doi: 10.1016/j.jad.2015.01.062. PubMed PMID: 25721612.

14. Janssen EP, Kohler S, Stehouwer CD, Schaper NC, Dagnelie PC, Sep SJ, et al. The Patient Health Questionnaire-9 as a Screening Tool for Depression in Individuals with Type 2 Diabetes Mellitus: The Maastricht Study. J Am Geriatr Soc. 2016;64(11):e201-e6. Epub 2016/10/27. doi: 10.1111/jgs.14388. PubMed PMID: 27783384.

15. Lamers F, Jonkers CC, Bosma H, Penninx BW, Knottnerus JA, van Eijk JT. Summed score of the Patient Health Questionnaire-9 was a reliable and valid method for depression screening in chronically ill elderly patients. J Clin Epidemiol. 2008;61(7):679-87. Epub 2008/06/10. doi: 10.1016/j.jclinepi.2007.07.018. PubMed PMID: 18538262.

16. Twist K, Stahl D, Amiel SA, Thomas S, Winkley K, Ismail K. Comparison of depressive symptoms in type 2 diabetes using a two-stage survey design. Psychosomatic medicine. 2013;75(8):791-7. Epub 2013/08/08. doi: 10.1097/PSY.0b013e3182a2b108. PubMed PMID: 23922402.

17. van Steenbergen-Weijenburg KM, de Vroege L, Ploeger RR, Brals JW, Vloedbeld MG, Veneman TF, et al. Validation of the PHQ-9 as a screening instrument for depression in diabetes patients in specialized outpatient clinics. BMC Health Serv Res. 2010;10(1):235. doi: 10.1186/1472-6963-10-235. PubMed PMID: 20704720; PubMed Central PMCID: PMCPMC2927590.

18. Zhang Y, Ting R, Lam M, Lam J, Nan H, Yeung R, et al. Measuring depressive symptoms using the Patient Health Questionnaire-9 in Hong Kong Chinese subjects with type 2 diabetes. J Affect Disord. 2013;151(2):660-6. Epub 2013/08/14. doi: 10.1016/j.jad.2013.07.014. PubMed PMID: 23938133.

19. Awata S, Bech P, Yoshida S, Hirai M, Suzuki S, Yamashita M, et al. Reliability and validity of the Japanese version of the World Health Organization-Five Well-Being Index in the context of detecting depression in diabetic patients. Psychiatry and clinical neurosciences. 2007;61(1):112-9. Epub 2007/01/24. doi: 10.1111/j.1440-1819.2007.01619.x. PubMed PMID: 17239048.

20. Krille S, Kulzer B, Reinecker H, Haak T, Hermanns N. Einflüsse von Psyche und Verhalten auf den Krankheitsverlauf (F54) bei Diabetes mellitus: Prävalenz und Screeningmethoden. Verhaltenstherapie & Verhaltensmedizin. 2008;29(4):323-35.

21. Yoshida S, Hirai M, Suzuki S, Awata S, Oka Y. Neuropathy is associated with depression independently of health-related quality of life in Japanese patients with diabetes. Psychiatry and clinical neurosciences. 2009;63(1):65-72. Epub 2008/12/11. doi: 10.1111/j.1440-1819.2008.01889.x. PubMed PMID: 19067994.
