# Supplementary material for: Diagnostic accuracy of depression questionnaires in adult patients with diabetes: A systematic review and meta-analysis
Source: PLoS One. 2019 Jun 20;14(6):e0218512. doi: 10.1371/journal.pone.0218512 (PMC6586329; doi:10.1371/journal.pone.0218512)
Supplement: S1 Text — (DOCX) [file pone.0218512.s007.docx]

**S1 Text. Risk of bias assessment - Signaling questions with interpretations**

The following signaling questions are used to assess the risk of bias according to the QUADAS-2^3^. If the answer on a question is “yes”, the risk of bias is low^3^.

**Domain 1 Patient selection**

- 1. Was a consecutive or random sample of patients enrolled?

*If this was not explicitly mentioned, but there was mentioned that all patients that visited a certain clinical in a certain time period, it was assumed that this was a consecutive sample (answered “Yes”).*

- 1. Was a case-control design avoided?

*Cases and controls based on the reference standard, not on the index test.*

- 1. Did the study avoid inappropriate exclusions?

*Inappropriate exclusions were among others: history of psychological disorder or depression, diabetes related comorbidities, (severe) diabetes complications*

**Domain 2 Index test**

- 1. Were the index test results interpreted without knowledge of the results of the reference standard?

*If the index test was given before the reference standard, this question was answered with “Yes”.*

- 1. If a threshold was used, was it pre-specified?

*If the aim of the study was to find the optimal threshold or the usual threshold was used, this question was answered with “Yes”.*

**Domain 3 Reference standard**

- 1. Is the reference standard likely to correctly classify the target condition?
     *If the clinical interview was not further specified, this question was answered with “Unclear”. This did not result in an unclear applicability concern because a clinical interview was seen as an appropriate reference standard.*
  2. Were the reference standard results interpreted without knowledge of the results of the index test?

*If this was not mentioned, this question was answered with “Unclear”. If interviewers were blinded partly, this question was answered with “No” because a part of the results had a high risk of bias.*

**Domain 4 Flow and Timing**

- 1. Was there an appropriate interval between index test and reference standard?

*A maximum of one month was seen as appropriate.*

- 1. Did all patients receive the same reference standard?

*Focus on “all” and “same”. If a random sample was selected to undergo a clinical interview, this question was answered with “Yes” because this probably did not affect the results. If this was not stated, but only “a sample” was selected, this question was answered with “No”.*

- 1. Were all patients included in the analysis?

*“All patients” was interpreted as the number of patients eligible for the study.
If a random sample was selected to undergo a clinical interview (reference standard), this question was answered with “Yes” because the potential bias on the results is low. If it was expected that the drop-out was selective: more serious ill patients dropped out or less serious cases, this question was answered with “No”.*

*If there was drop-out, but there was stated that the patients did not differ on demographics, this question was answered with “Yes”. If this is not mentioned, as “Unclear”. But if this was not mentioned and the drop-out rate was > 20%, this question was answered with “No”.*
