# Supplementary figures and images for: Diagnostic accuracy of depression questionnaires in adult patients with diabetes: A systematic review and meta-analysis
Source: PLoS One. 2019 Jun 20;14(6):e0218512. doi: 10.1371/journal.pone.0218512 (PMC6586329; doi:10.1371/journal.pone.0218512)

**S1 Fig. SROC plots of the (A) CES-D (≥16), (B) PHQ-9(≥10) and (C) PHQ-9 algorithm**

| ***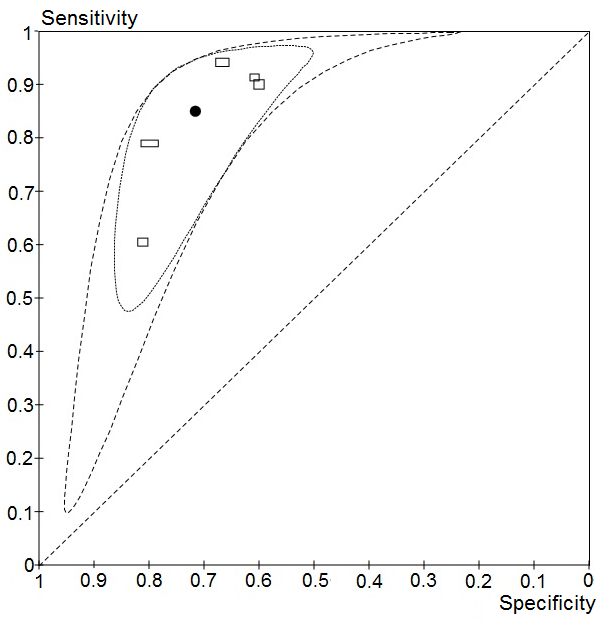***  **A** | ***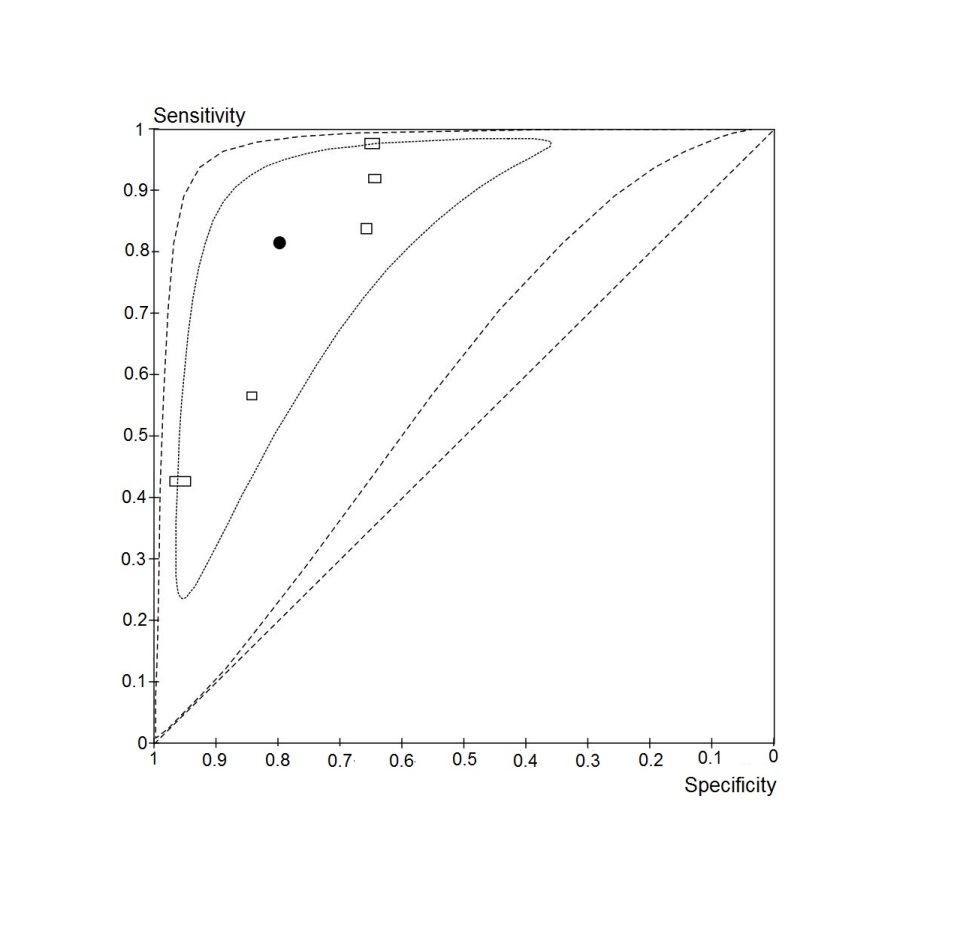***  **B** | ***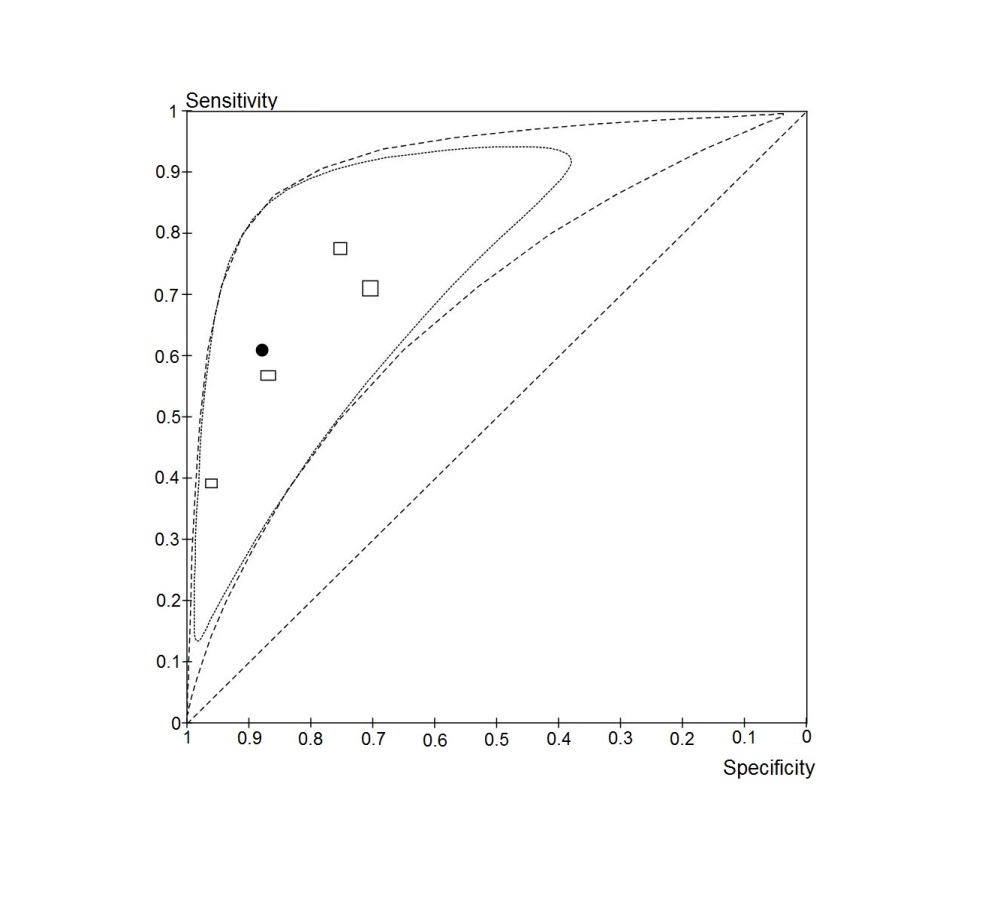***  **C** |
| --- | --- | --- |
| 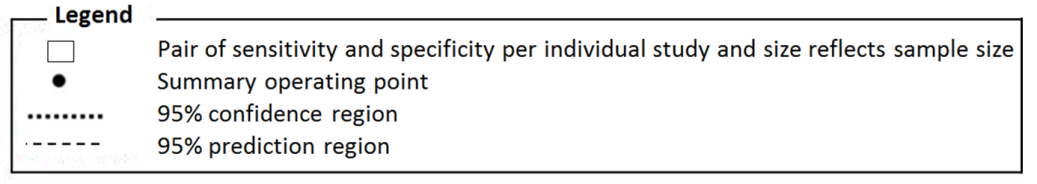 | | |
|  | | |

Supplement: S1 Fig — SROC plots of the (A) CES-D (≥16), (B) PHQ-9(≥10) and (C) PHQ-9 algorithm. (DOCX) [file pone.0218512.s008.docx]
